# Staphylococcal enterotoxin-like X (SElX) is a unique superantigen with functional features of two major families of staphylococcal virulence factors

**DOI:** 10.1371/journal.ppat.1006549

**Published:** 2017-09-07

**Authors:** Ries J. Langley, Yi Tian Ting, Fiona Clow, Paul G. Young, Fiona J. Radcliff, Jeong Min Choi, Richard P. Sequeira, Silva Holtfreter, Heather Baker, John D. Fraser

**Affiliations:** 1 School of Medical Sciences, and The Maurice Wilkins Centre for Molecular Biodiscovery, the University of Auckland, Auckland, New Zealand; 2 School of Biological Sciences, and The Maurice Wilkins Centre for Molecular Biodiscovery, the University of Auckland, Auckland, New Zealand; Columbia University, UNITED STATES

## Abstract

*Staphylococcus aureus* is an opportunistic pathogen that produces many virulence factors. Two major families of which are the staphylococcal superantigens (SAgs) and the Staphylococcal Superantigen-Like (SSL) exoproteins. The former are immunomodulatory toxins that induce a Vβ-specific activation of T cells, while the latter are immune evasion molecules that interfere with a wide range of innate immune defences. The superantigenic properties of Staphylococcal enterotoxin-like X (SElX) have recently been established. We now reveal that SElX also possesses functional characteristics of the SSLs. A region of SElX displays high homology to the sialyl-lactosamine (sLacNac)-specific binding site present in a sub-family of SSLs. By analysing the interaction of SElX with sLacNac-containing glycans we show that SElX has an equivalent specificity and host cell binding range to the SSLs. Mutation of key amino acids in this conserved region affects the ability of SElX to bind to cells of myeloid origin and significantly reduces its ability to protect *S*. *aureus* from destruction in a whole blood killing (WBK) assay. Like the SSLs, SElX is up-regulated early during infection and is under the control of the *S*. *aureus* exotoxin expression (Sae) two component gene regulatory system. Additionally, the structure of SElX in complex with the sLacNac-containing tetrasaccharide sialyl Lewis X (sLeX) reveals that SElX is a unique single-domain SAg. In summary, SElX is an ‘SSL-like’ SAg.

## Author summary

The ability of *Staphylococcus aureus* to cause disease can be attributed to the wide range of toxins and immune evasion molecules it produces. The 25-member superantigen (SAg) family of toxins disrupts adaptive immunity by activating large proportions of T cells. In contrast, the structurally-related 14-member Staphylococcal Superantigen-Like (SSL) family inhibits a wide range of innate immune functions. We have discovered that the SAg staphylococcal enterotoxin-like X (SElX) has the sialylated-glycan-dependent active site found in a sub-family of SSLs. Through this site it possesses the ability to affect host innate immunity defences. By solving the X-ray crystal structure of SElX we have also discovered that SElX is a unique single-domain SAg. While it retains a typical β-grasp domain, it lacks the OB-fold domain that is present in all other staphylococcal SAgs.

## Introduction

*Staphylococcus aureus* is a serious human pathogen responsible for a large proportion of hospital acquired infections and of additional major concern, an increasing cause of community-associated antibiotic resistant infections [1, 2]. Predominantly found in the anterior nares, throat, and skin it persistently colonises ~30% of the population with anywhere from 50–80% of individuals carrying it at any particular time point [3]. *S*. *aureus* is an opportunistic pathogen and although considered commensal, in many situations is capable of overcoming the host barrier defences to infect potentially any part of the body [4, 5]. The ability of *S*. *aureus* to so effectively cause infection is a consequence of the myriad of virulence factors it produces. Toxins, enzymes, adhesion molecules, and immune evasion molecules allow the bacterium to first invade the host and then to get established and avoid destruction by the immune system [6, 7]. One such class of virulence factor, the superantigen (SAg), has been extensively studied over the past few decades (reviewed in [8–18]. These toxins are known to cause staphylococcal toxic shock syndrome and staphylococcal food poisoning, and have been implicated in a number of conditions including sepsis, endocarditis, and pneumonia. The staphylococcal SAgs form a large family of related toxins with the SAgs found in many streptococcal species, most notably *Streptococcus pyogenes*. A critical SAg involvement in establishing infection of its natural niche has been discovered for *S*. *pyogenes* [19], and a role for SAgs in promoting survival of *S*. *aureus* during infection has been identified [20].

The bacterial SAgs of *S*. *aureus* and *S*. *pyogenes* are a family of secreted toxins of around 20–30 kD that are most notably known for causing the toxic shock syndromes associated with these pathogens. By simultaneously binding major histocompatibility complex (MHC) class II on antigen presenting cells and the T cell receptor (TcR) in a Vβ-specific manner they are able to activate large proportions of T cells to produce pro-inflammatory cytokines. It is the ensuing ‘cytokine storm’ that is responsible for the symptoms of shock and organ failure that result. Currently there are 25 known staphylococcal and 11 streptococcal SAgs many of which have been crystallized alone or in complex with MHC class II, TcR, or both [21, 22]. These SAgs share a common fold consisting of two highly stable domains, the N-terminal OB-fold domain and the C-terminal β-grasp domain, that are separated by a long, partially solvent-accessible central α-helix [23]. Interestingly, this same protein fold is also found in the functionally unrelated, 14-member family of Staphylococcal Superantigen-Like toxins (SSLs) [24]. Although the staphylococcal and streptococcal SAgs and the SSLs share limited sequence homology they can be identified by the two highly conserved PROSITE “family signature motifs” Y-G-G-[LIV]-T-X(4)-N (Prosite entry PS00277) and K-X(2)-[LIVF]-X(4)-[LIVF]-D-X(3)-R-X(2)-L-X(5)-[LIV]-Y (PS00278) [25].

The Staphylococcal Superantigen-Like (SSL) family of proteins are related to the SAgs by sequence and structure [26]. The SSLs however do not function as superantigens, rather are involved in blocking various aspects of host immunity. For instance, SSL7 binds to IgA and complement C5 to prevent C5a-mediated chemotaxis of inflammatory myeloid cells and C5-dependent microbial killing [27, 28]. SSL10 binds to human IgG1 to inhibit the phagocytosis and complement activation mediated by this important immunoglobulin [29, 30]. SSL3 binds to toll-like receptor 2 and inhibits its capacity to signal in response to pathogen associated molecular patterns [31, 32]. SSL5 and SSL11 bind to P-selectin glycoprotein ligand-1 (PSGL-1) via a highly conserved site with specificity for sialylated glycans that contain the minimal conserved trisaccharide, sialyl-lactosamine (sLacNac = NeuAcα2-3Galβ1-4GlcNAc). The interaction with PSGL-1 prevents P-selectin mediated immune cell recruitment. [33, 34]. They share this binding site with SSLs 2–4, and SSL6 [35]. This conserved site has been implicated in the interactions of the sialylated-glycan binding SSLs with a diverse range of additional host glycoproteins that include the Fc receptor for IgA, the glycosylated N-terminal region of G protein-coupled receptors, platelet glycoproteins (GPIbα, GPIIb-IIIa, and GPVI), CD47, and matrix metalloproteinase-9 [34–40].

The recently discovered staphylococcal SAg, SElX, is unusual in that it is chromosomally located and thus found in all strains of *S*. *aureus* with the exception of clonal complex 30 [41]. It possesses an entirely unique N-terminus of no known homology that is much shorter in residues than the OB-fold domain of other SAgs. The C-terminal half of SElX however displays amino acid similarity with the β-grasp domain of both the SAgs and the SSLs. Recently SElX, like SSL5 and SSL11, was shown to bind PSGL-1 in a sialylated-glycan-dependent manner to inhibit its interaction with P-selectin [38].

Here we present functional and structural evidence that SElX is an ‘SSL-like’ SAg. It has specificity for sLacNac and interacts with myeloid cells in a sialylated glycan-dependent manner to inhibit host defences. Additionally, X-ray crystallography of SElX reveals a unique structural variation from the typical SAg architecture, with the complete omission of an OB-fold domain.

## Results

### SElX has homology to the glycan binding subfamily of SSLs

**Fig 1:**
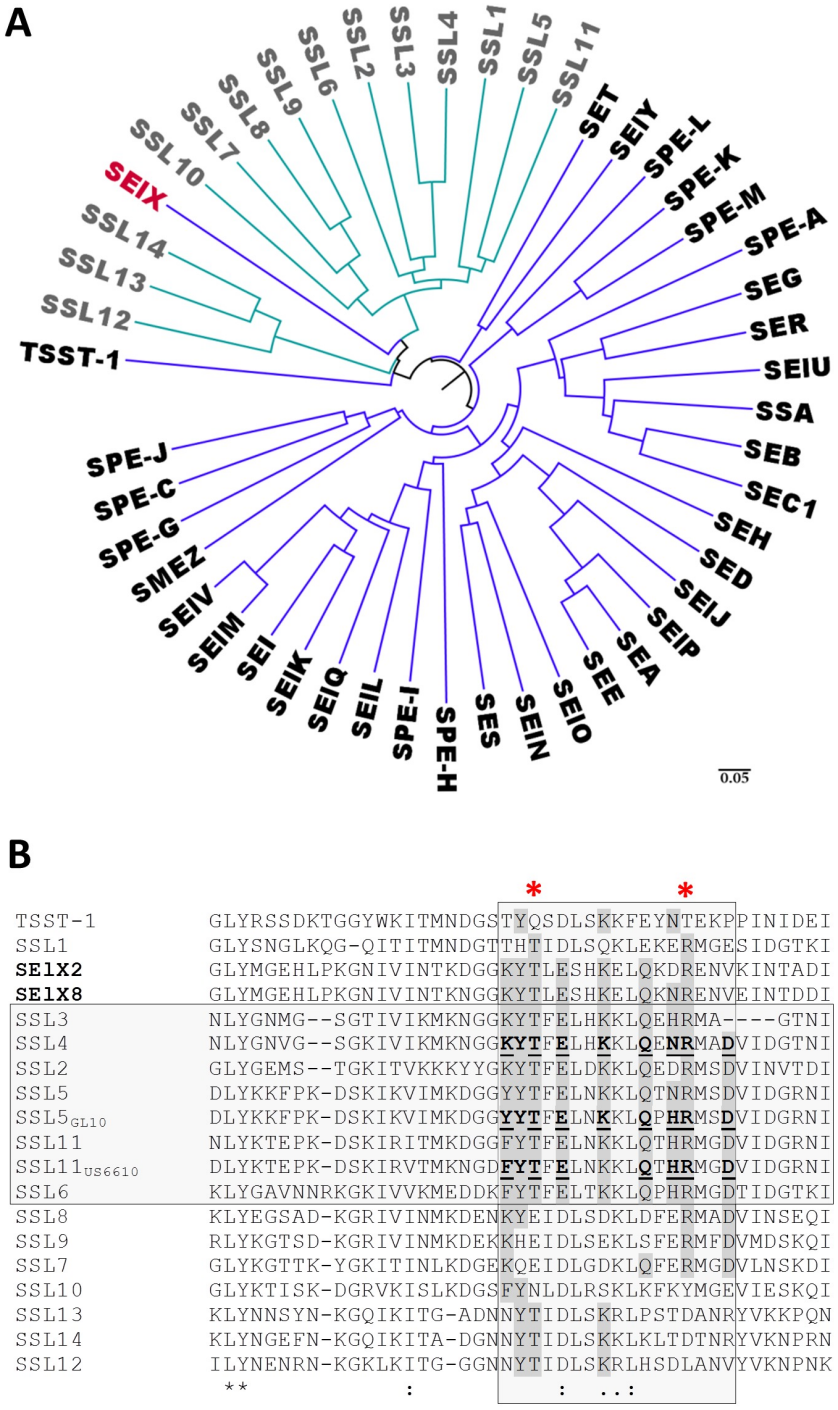
Comparison of SElX with SAgs and SSLs. (A) Phylogenetic analysis of the SAgs and SSLs of *S*. *aureus*. The Phylogenetic tree created using FigTree (v1.4.2) from an amino acid alignment of the staphylococcal SAgs and SSLs generated using Clustal Omega (EMBL-EBI). SAgs are shown in black text and the SSLs in grey text. SElX is shown in red. (B) Amino acid sequence alignment of the two SElX variants used in this study, SElX2 and SElX8 (in bold), with the SSLs in the region of the sialylated glycan-dependent binding site. The glycan binding SSL subfamily is highlighted by the horizontal grey box and the region of the 17 amino acid glycan binding site is highlighted by the vertical grey box. Residues that have been experimentally determined to interact with the sialylated glycan are shown in bold type with those that hydrogen bond to the glycan underlined. Residues with homology to these amino acids are highlighted in dark grey. The conserved Threonine (T) and Arginine (R) residues mutated to affect sialylated glycan binding are indicated by the red asterisks.

SElX has been reported to display highest homology to TSST-1 and SSL7 [41]. Phylogenetic analysis indicates that SElX is more closely related to the SSL family of immune evasion proteins than to the bacterial superantigen family (Fig 1A). Additionally SElX shows closer sequence conservation to the SSLs in a region of the central α-helix that makes up the PROSITE signature sequence PS00278. In SElX the sequence KELD has higher identity to the SSL consensus sequence of KE(L/I)D than the consensus sequence of the SAgs QE(L/I/V)D. The Lysine (K) of this motif is absolutely conserved in the SSLs whereas the Glutamine (Q) is almost entirely conserved in the SAgs (S1 Fig). Amino acid sequence alignment with the SSLs reveals that SElX possesses significant identity to SSLs 2–6 and SSL11 in the region of conservation that describes the glycan binding site of this SSL subfamily (Fig 1B) [34, 35, 42]. Of the seventeen residues that define the conserved glycan-binding site, SElX displays higher homology than SSLs from outside the glycan-binding subfamily. In particular, residues known to interact with sialylated glycans are highly conserved in SElX (Fig 1B). Furthermore, no homology with residues that form the α-chain or β-chain MHC class II binding sites can be seen upon alignment of SElX with the other bacterial SAgs (S1 Fig). For this reason the characterization of SElX as a novel SSL-like SAg was performed. Two variants of SElX were analysed, one being SElX2 cloned from the CC8 strain Newman and the other SElX8 cloned from strain JSNZ. JSNZ is a mouse-adapted strain of *S*. *aureus* isolated from preputial gland abscesses during a severe outbreak among male C57BL/6 mice [43]. This strain is from the multilocus sequence type ST88 and encodes no other identifiable SAgs other than SElX.

### SElX binds to sialylated glycans

**Fig 2:**
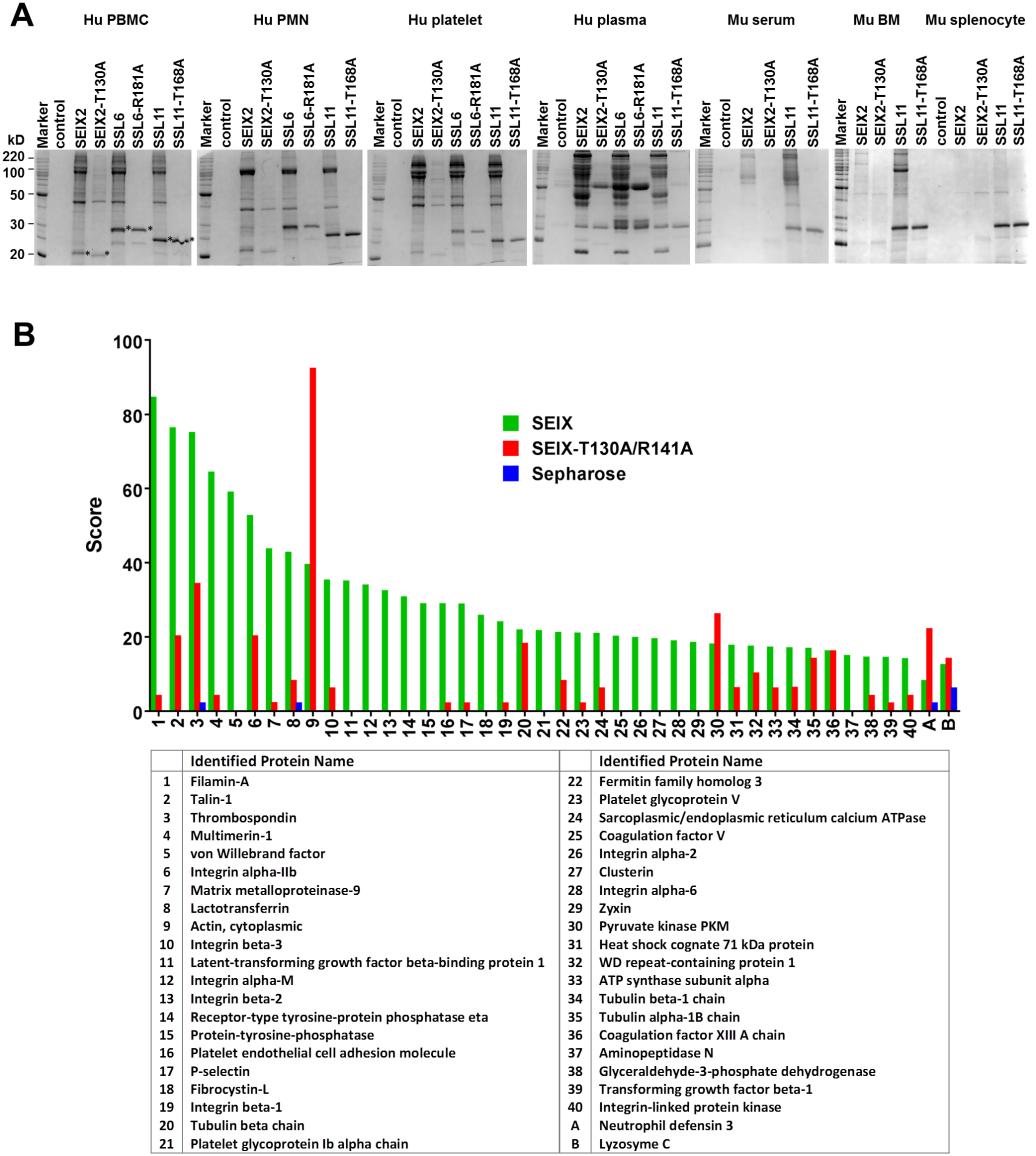
Analysis of host binding by affinity precipitation. (A) SDS-PAGE (12.5%) run under reducing and denaturing conditions of proteins from the various indicated human and mouse sources isolated by affinity with SElX2, SElX2-T130A, SSL6, SSL6-R181A, SSL11, or SSL11-T168A coupled to sepharose. Sepharose alone (control) was used as a control for non-specific binding. * indicates SELX/SSL that has dissociated from the sepharose. Marker is BenchMark Protein Ladder (Life Technologies). (B) The 40 top scoring leukocyte proteins identified by SElX-sepharose affinity binding are shown by descending rank in the bar graph and listed in order in the accompanying table. The ranking is based on the Unused Score (taken from S1 Table) given to each uniquely identified protein as calculated by the mass spectrometry analysis software ProteinPilot 5.0 (AB Sciex Pte. Ltd). The Unused Score indicates how much of the Total Score is unique to the particular protein hit. The Total Score is the sum of the Contrib values (contrib = the highest scoring peptide match for a peptide sequence) and determines the overall confidence for the protein identification. A and B are the Unused Scores of additional proteins identified in both the SElX-T130A/R141A and sepharose control samples with their corresponding Unused Scores from the SElX sample.

To determine if SElX is a glycan binding protein, host protein binding assays were performed to compare SElX with the known glycan binding proteins SSL4, SSL6, and SSL11. The glycan binding SSLs display broad binding capacity for plasma and myeloid cell glycoproteins [33–40, 42]. Recombinant SElX2, SSL6, and SSL11 coupled to sepharose were used to isolate interacting proteins from human peripheral blood mononuclear cells (PBMC), peripheral blood polymorphonuclear cells (PMN), platelets, and plasma. Binding to mouse serum, and splenocyte and bone marrow (BM) derived cell lysates was also compared. The binding profiles of proteins bound by SElX2 in each instance were very similar to those of SSL6 and SSL11 (Fig 2A). Previously, mutagenesis of the conserved Threonine (T) or Arginine (R) (indicated by the red asterisks in Fig 1B) in the glycan binding SSLs has resulted in greatly diminished capacity for carbohydrate-dependent interactions [34, 35, 42]. Mutation of the equivalent residue Threonine 130 (T130) or Arginine 141 (R141) to Alanine in SElX2 resulted in a significant reduction in host protein binding that is similar to mutating the equivalent Threonine in SSL11 or Arginine in SSL6 (Fig 2A and S2 Fig). Additionally, SElX displayed a comparable energy- and glycan binding site-dependent binding to neutrophils as has previously been reported for SSL4 and SSL11 [34, 42] (S2 Fig).

**Fig 3:**
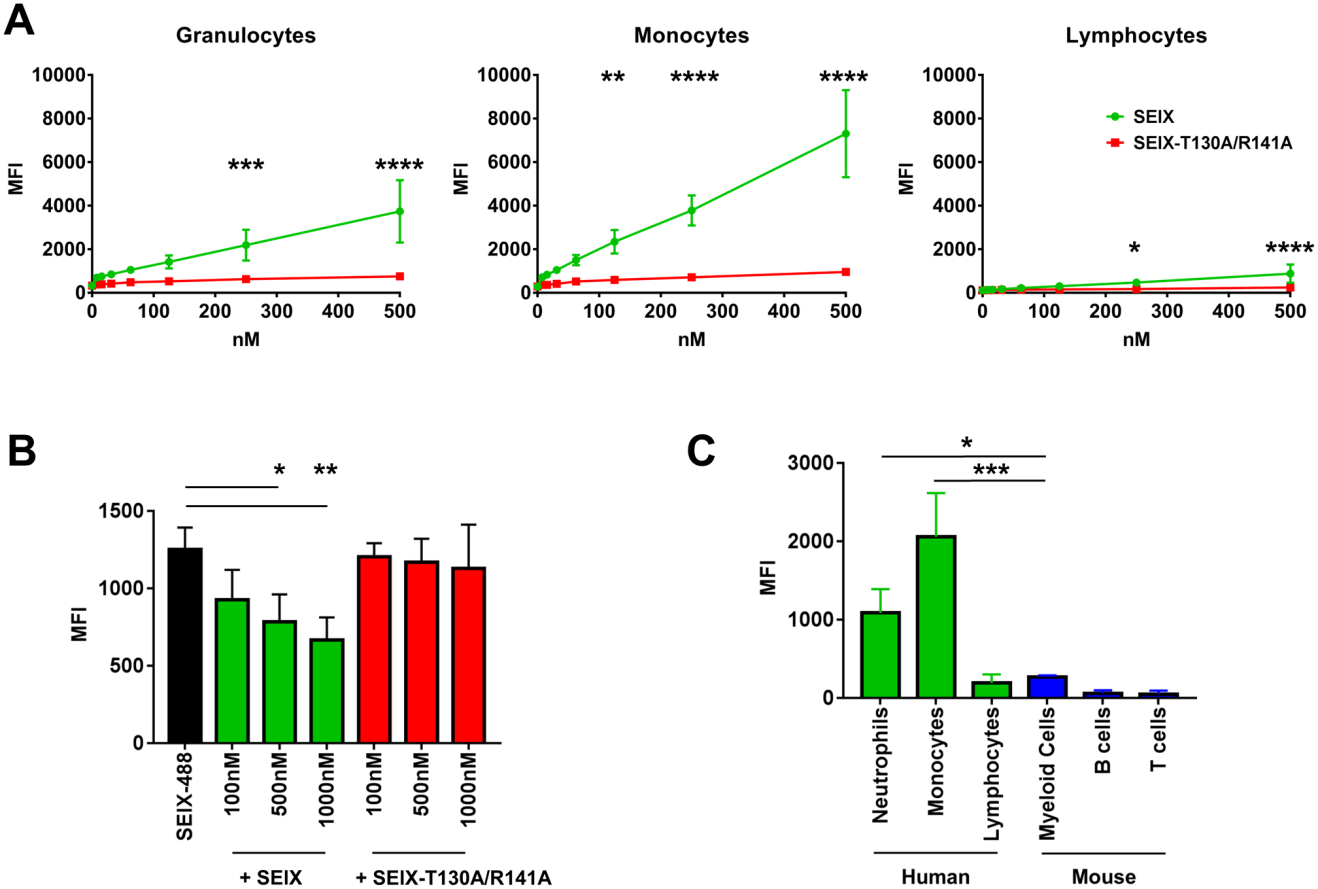
Analysis of host binding by flow cytometry. (A) Median fluorescence intensity (MFI) of a two-fold dilution series of SElX-488 (green line) or SElX-T130A/R141A (red line) binding to human leukocytes with cell populations gated as granulocytes, monocytes, and lymphocytes based on size and granularity. Each data point represents the mean ± SD of three separate experiments using three individual donors. Comparison of the two proteins binding each cell population was performed in Graphpad Prism using two way analysis of variance (ANOVA) (p<0.0001) with Sidak’s multiple comparisons test: * p = 0.0336; ** p = 0.0037; ***p = 0.0005; **** p < 0.0001. (B) Binding of 100nM SElX-488 to human granulocytes (black bar) and in the presence of increasing concentrations of SElX (Green bars) or SElX-T130A/R141A (red bars). The MFI is the mean ± SD of three experiments performed using three separate human donors. The column data were compared by two-tailed paired t-tests: *p = 0.0333; ** p = 0.0038. (C) Comparison of the binding of 100nM SElX-488 to human and mouse leukocyte populations. The MFI is the mean ± SD of three experiments performed using three separate human donors or two experiments on n = 1 mouse per experiment and is the MFI (SElX-488 stained cells) minus MFI (unstained control population). Data compared by one way ANOVA (p<0.0001) with Tukey’s multiple comparisons test: * p = 0.0189; *** p = 0.0005.

Identification of host leukocyte proteins bound by SElX and its glycan-binding site mutant SElX-T130A/R141A was performed using liquid chromatography—tandem mass spectrometry (LC-MS/MS) on lysate proteins captured by affinity precipitation using the immobilized SElX variants. The top scoring proteins identified are shown in Fig 2B and full data on the identified proteins and their functions is available in S1 Table. The mass spectrometry data show that many of the leukocyte proteins that are bound by SElX are integrins. Other adhesion molecules such as P-selectin and PECAM-1 feature in this list. Several of the SElX-targeted proteins are cytoplasmic in origin and are predominantly granule proteins or are associated with the cytoskeletal network, with roles linking the cytoskeleton to surface receptors. Furthermore, a large number of the identified proteins are involved in coagulation. The predominance of proteins identified to interact with SElX-T1301/R141 were intracellular and associated with the cytoskeleton with the integrin alpha IIb and beta 3 chains the only exception. Flow cytometry of recombinant SElX2 conjugated to Alexa Fluor 448 (SElX-448) revealed that SElX bound to human granulocytes, monocytes, and weakly to lymphocytes whereas negligible binding was seen using the glycan-binding site mutant SElX-T130A/R141A (Fig 3A). To further support the glycan-binding site dependency of cell binding, competition for the binding of SElX-488 to neutrophils using increasing concentrations of SElX or SElX-T130A/R141A was performed. The cell surface interaction of SElX-488 could be inhibited in a dose dependent manner with SElX whereas the glycan-binding site mutant showed no significant inhibition at any of the concentrations used (Fig 3B). The host specificity of SElX binding was compared using human peripheral blood leukocytes and mouse splenic and bone marrow leukocytes. The SElX8-488 variant from the mouse-adapted strain JSNZ was used for this analysis and showed a greater capacity for binding to human cells (Fig 3C).

Recombinant SElX2 was analysed for carbohydrate binding to a glycan array that contained 611 mammalian glycan targets by the Consortium for Functional Glycomics. The array screening confirmed that SElX2 bound glycans containing the trisaccharide sialyl-lactosamine (sLacNac = Neu5Aca2-3Galb1-4GlcNAc). Several of the strongly bound glycans terminated in the sLacNac-containing tetrasaccharide sialyl Lewis X (sLeX = Neu5Acα2-3Galβ1-4(Fucα1–3)GlcNAc) (Table 1 and S3 Fig).

**Table 1:** Ten strongest SElX binding glycans from the glycomics consortium array.

glycan name	Average RFU	StDev
Neu5Aca2-3Galb1-4(Fuca1-3)GlcNAcb1-3Galb1-4(Fuca1-3)GlcNAcb1-3Galb1-4(Fuca1-3)GlcNAcb-Sp0	3561	126
Neu5Aca2-3Galb1-4(Fuca1-3)GlcNAcb1-3Galb-Sp8	3526	100
Neu5Aca2-3Galb1-4(Fuca1-3)GlcNAcb1-3Galb1-4GlcNAcb-Sp8	2416	52
Neu5Aca2-3Galb1-4GlcNAcb1-6(Neu5Aca2-3Galb1-4GlcNAcb1-3)GalNAca-Sp14	2237	277
Neu5Aca2-3Galb1-4GlcNAcb1-3GalNAc-Sp14	1623	59
Neu5Aca2-3Galb1-4(Fuca1-3)GlcNAcb1-2Mana-Sp0	1355	126
Neu5Aca2-3Galb1-4GlcNAcb1-3Galb1-4GlcNAcb-Sp0	1326	88
Neu5Aca2-3Galb1-4GlcNAcb1-3Galb1-4GlcNAcb1-6(Neu5Aca2-3Galb1-4GlcNAcb1-3Galb1-4GlcNAcb1-3)GalNAca-Sp14	1231	22
Neu5Aca2-3Galb1-4GlcNAcb1-3Galb1-3GlcNAcb-Sp0	1219	14
Neu5Aca2-3Galb1-4GlcNAcb1-2Mana1-6(Neu5Aca2-3Galb1-4GlcNAcb1-2Mana1-3)Manb1-4GlcNAcb1-4GlcNAcb-Sp12	1162	41

**Fig 4:**
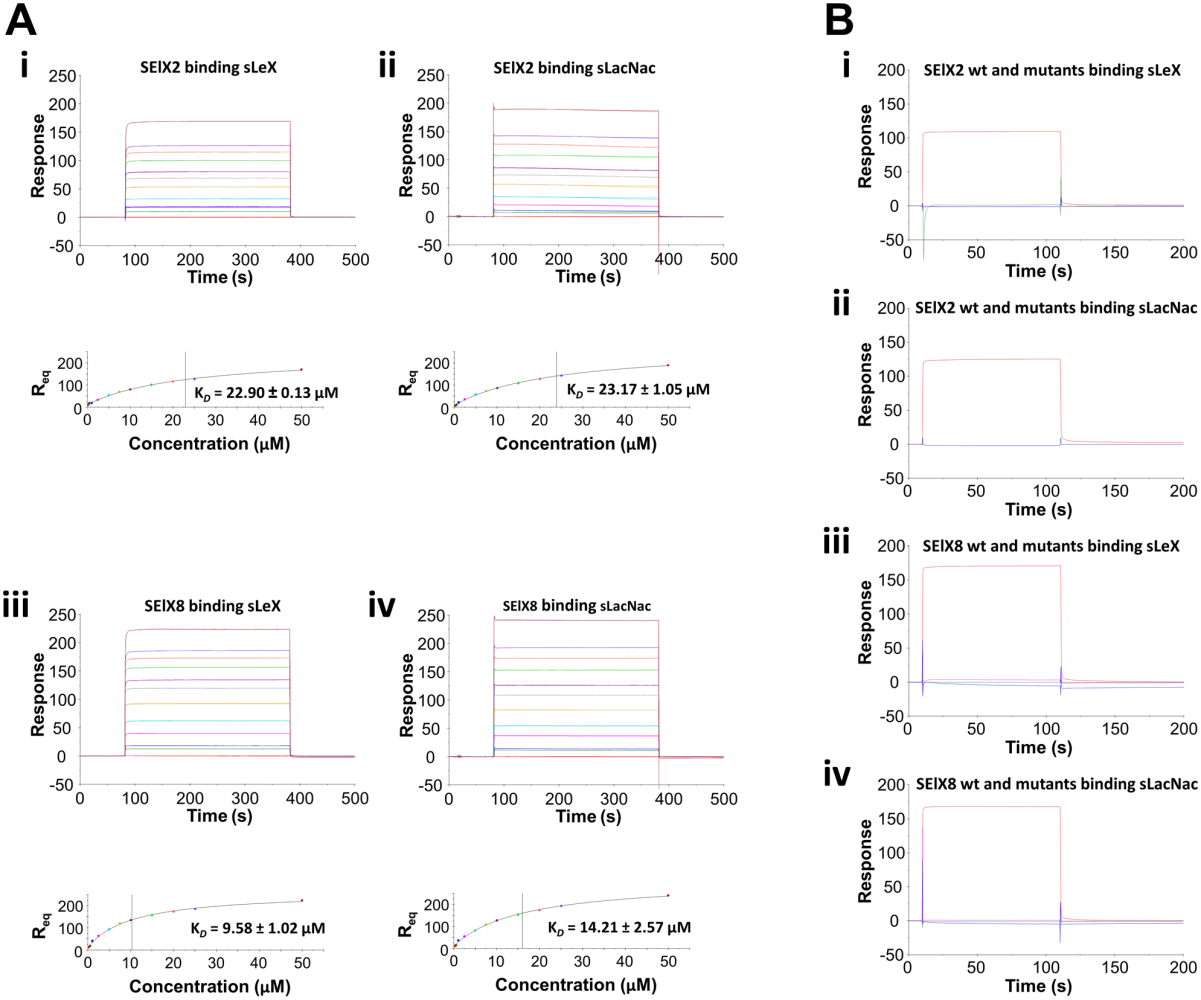
Determination of SElX2 and SElX8 binding to sLeX and sLacNac by surface plasmon resonance. (A) Quantitative measure of SElX2 and SElX8 binding to sLeX and sLacNac. Binding responses at equilibrium (Req) are shown against the concentration and fitted to a steady-state affinity binding model to calculate an equilibrium affinity constant (KD). (i) sLeX sensor chip binding and equilibrium binding analysis of 0.25 to 50 μM SElX2 in duplicate. (ii) sLacNac sensor chip binding and equilibrium binding analysis of 0.25 to 50 μM SElX2 in duplicate. (iii) sLeX sensor chip binding and equilibrium binding analysis of 0.25 to 50 μM SElX8 in duplicate. (iv) sLacNac sensor chip binding and equilibrium binding analysis of 0.25 to 50 μM SElX8 in duplicate. (B) Comparison of SElX and its glycan-binding mutants to sLeX and sLacNac. (i) sLeX sensor chip binding SElX2 (red), SElX2-T130A (pink), SElX2-R141A (green), and SElX2-T130A/R141A (blue) at 20 μM. (ii) sLacNac sensor chip binding SElX2 (red), SElX2-T130A (pink), SElX2-R141A (green), and SElX2-T130A/R141A (blue) at 20 μM. (iii) sLeX sensor chip binding SElX8 (red), SElX8-T130A (pink), SElX8-R141A (green), and SElX8-T130A/R141A (blue) at 20 μM. (iv) sLacNac sensor chip binding SElX8 (red), SElX8-T130A (pink), SElX8-R141A (green), and SElX8-T130A/R141A (blue) at 20 μM. The plots shown are representative of three independent experiments where each experiment was performed in duplicate. The affinity (K_*D*_) values are expressed as mean ± SD of the repeats.

Surface plasmon resonance (SPR) was employed to study the interaction between SElX and its sialylated target ligands. The affinities of recombinant SElX2 and SElX8 for sLeX and its core trisaccharide sLacNac (a subcomponent of sLeX lacking the terminal fucose) were determined by passing a concentration series of the highly purified proteins over the immobilized carbohydrates (Fig 4A). Equilibrium dissociation constants (K_*D*_) were calculated from the equilibrium binding curves acquired from both SElX2 and SElX8 binding to sLeX and sLacNac. The K_*D*_ of SElX2 was determined to be 22.90 ± 0.13 μM for sLeX, and 23.17 ± 1.05 μM for sLacNac. SElX8 bound sLeX and sLacNac with K_*D*_’s of 9.58 ± 1.02 μM and 14.21 ± 2.57 μM, respectively. Negligible binding was observed for the T130A, R141A, and T130A/R141A mutants of SEIX2 and SEIX8 to either sLeX or sLacNac (Fig 4B).

### SElX possesses a unique structure

**Fig 5:**
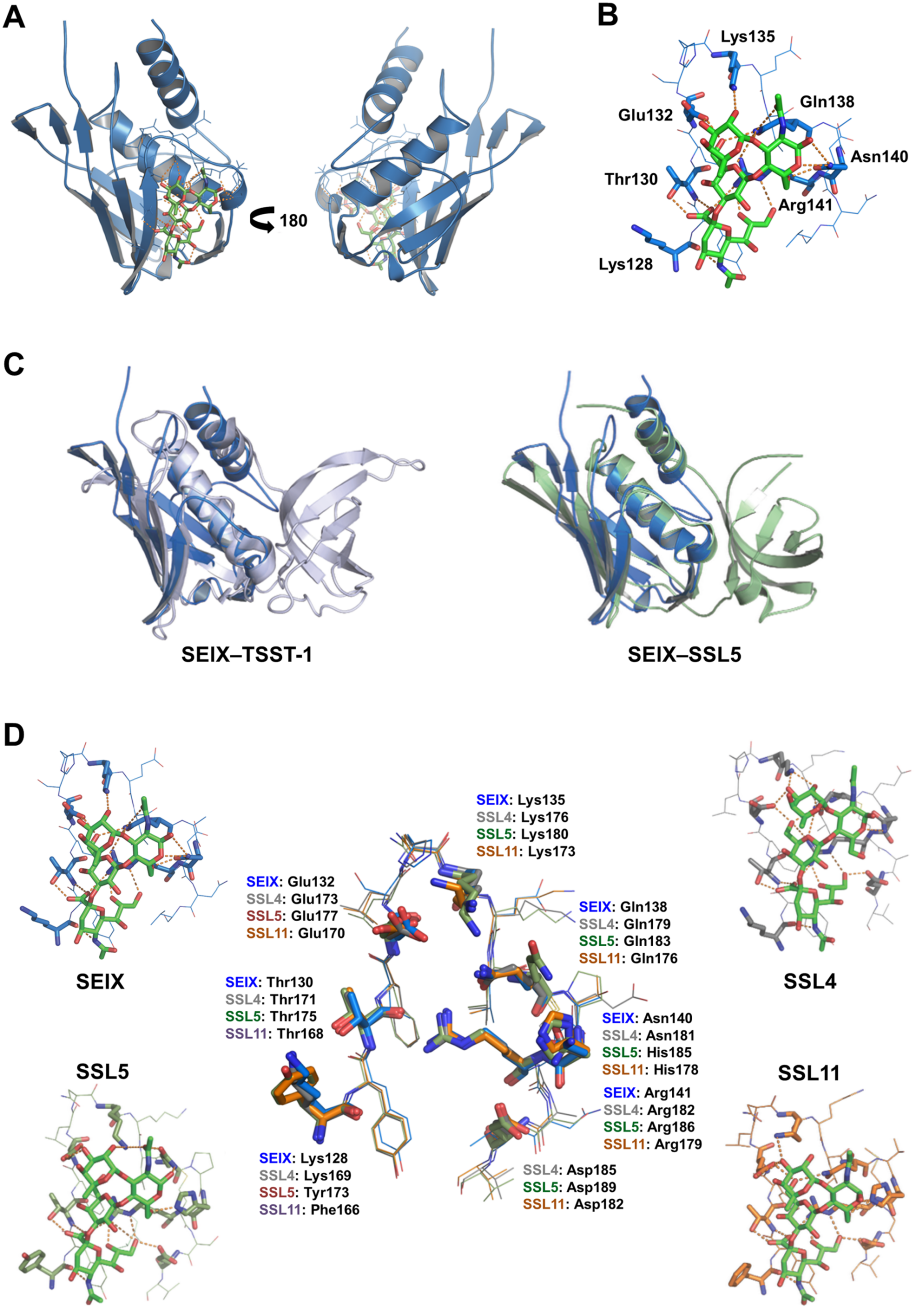
The structural analysis of SElX. (A) crystal structure of SElX8 (cyan) in complex with sLeX (green) shown from the left of the glycan binding site (left panel) and from the right of the glycan binding site (right panel). (B) The sialylated glycan-binding site of SElX8 (blue) showing the residues that hydrogen-bond (yellow dotted lines) with sLeX (green). The side chains that interact with sLeX are labelled and shown in bold. The components of sLeX are labelled as follows: N-Acetylneuraminic Acid (S); galactose (G); fucose (Fuc); and N-Acetylglucosamine (N). (C) Structural overlay of SElX8 (blue) with the SAg TSST-1 (silver) and SSL5 (sage). (D) Comparison of sialyl Lewis X (sLeX) (in green) bound in the glycan binding sites of SElX8 (blue), SSL4 (silver), SSL5 (sage), and SSL11 (orange). The side chains of residues that hydrogen-bond with sLeX are shown in bold. An overlay of these binding sites (centre) shows the conservation of residues that interact with sLeX.

The crystal structure of SEIX8 was determined in complex with sLeX. The protein structure was solved by molecular replacement with a partial model of SSL4 (PDB: 4DXG) using residues 130–200 and was refined at 1.66 Å (Table 2). The C-terminal domain of SEIX8 (residues N61–I161) adopts the β-grasp fold typical of the classical SAgs and members of the SSL family. The first 21 residues and the last 3 residues of the mature protein were undefined in the crystal structure, lacking electron density. The first structured residue N22 defines the start of the α-helix that sits atop the rear of the β-grasp C-terminal domain (Fig 5A). An extended loop, which is stabilized by extensive hydrogen bonds, links the bottom of this helix directly with the first β strand of the β-grasp domain. (Fig 5A). This linker region is predominantly unstructured, containing a β-hairpin and a series of β-turns as it packs across the side of the β-grasp domain. It completely replaces the typical OB-fold domain, revealing SElX to be a unique single-domain SAg.

**Table 2:** Data collection and refinement statistics parameters.

**Data collection**	
Space group	P65
Cell dimensions	
*a*, *b*, *c* (Å)	90.00 90.00 120.00
a, b, g (°)	92.14 92.14 53.42
Resolution (Å)	18.85–1.66 (1.75–1.66)
*R*_merge_	0.151 (1.94)
*I*/s*I*	17.9(1.5)
Completeness (%)	94.4 (87.6)
Redundancy	15.6 (14.9)
CC1/2	0.964
Mosacity	0.24
**Refinement**	
Resolution (Å)	46.06–2.40
*R*_work/_ *R*_free_	0.1882/ 0.2143
Total Observation	447883(57104)
No. unique reflections	28745 (3843)
Rms Bond Length	0.0092
Rms Bond Angle	1.3846
Rms Chiral Volume	0.0759

### Interaction of SElX with sLeX

The binding site for sLeX is a V-shaped depression in the side of the β-grasp domain, which is formed by residues from a β-strand, the opposing helix (a 3_10_-helix), and the irregular polypeptide loop that links them (Fig 5A). This region is on the opposite side of the β-grasp to the N-terminus linker loop. Seven residues lining the sides of the depression hydrogen bond directly to sLeX (Fig 5B). These are the side-chains of T130, E132, K135, Q138, N140, and R141, and the main chain carbonyl of K128. K128, T130, and R141 form an extensive network of hydrogen bonds with the sialic acid (S) of sLeX while Y129 participates in a hydrophobic interaction with this moiety. E132 hydrogen bonds with the galactose (G) and fucose (F) of sLeX, and with the side chain of K135, while K135 makes one additional hydrogen bond to the fucose. Extensive hydrogen bonding occurs between Q138 and both the galactose and *N*-acetylglucosamine (GlcNAc or N) sugars with further hydrogen bonding to GlcNAc provided by N140. An additional three waters make intermediate contacts directly between sLeX and SEIX. R141 makes an extensive network of hydrogen bonds to T130, Q138, and V144 across the floor of the binding site.

### Structural comparison of SElX with TSST-1 and the SSLs

The most striking feature of SElX is the absence of the ubiquitous N-terminal SAg/SSL OB-fold domain. Structural comparison with TSST-1 and SSL5 reveals that despite this deleted domain, the N-terminal α-helix of SElX is spatially conserved (Fig 5C) and just like the SAgs and SSLs it packs against the back of the β-grasp domain.

The sLeX binding site of SElX shows high structural conservation with the 17-residue glycan-binding region of the SSLs. The RMSDs (all atoms) are 0.73927 Å (over 152 atoms), 0.900455 Å (over 155 atoms), and 0.78852 Å (over 154 atoms) between SElX and SSL4, SSL5, and SSL11, respectively (Fig 5D). Indeed, the binding of sLeX is almost entirely conserved with the sLeX binding of SSL4 [42], SSL5 [35], and SSL11 [34]. The only exception is the loss of a conserved aspartic acid (V144 in SEIX), which in the SSL’s interacts with the sialic acid moiety of the glycan (Fig 5D).

**Fig 6:**
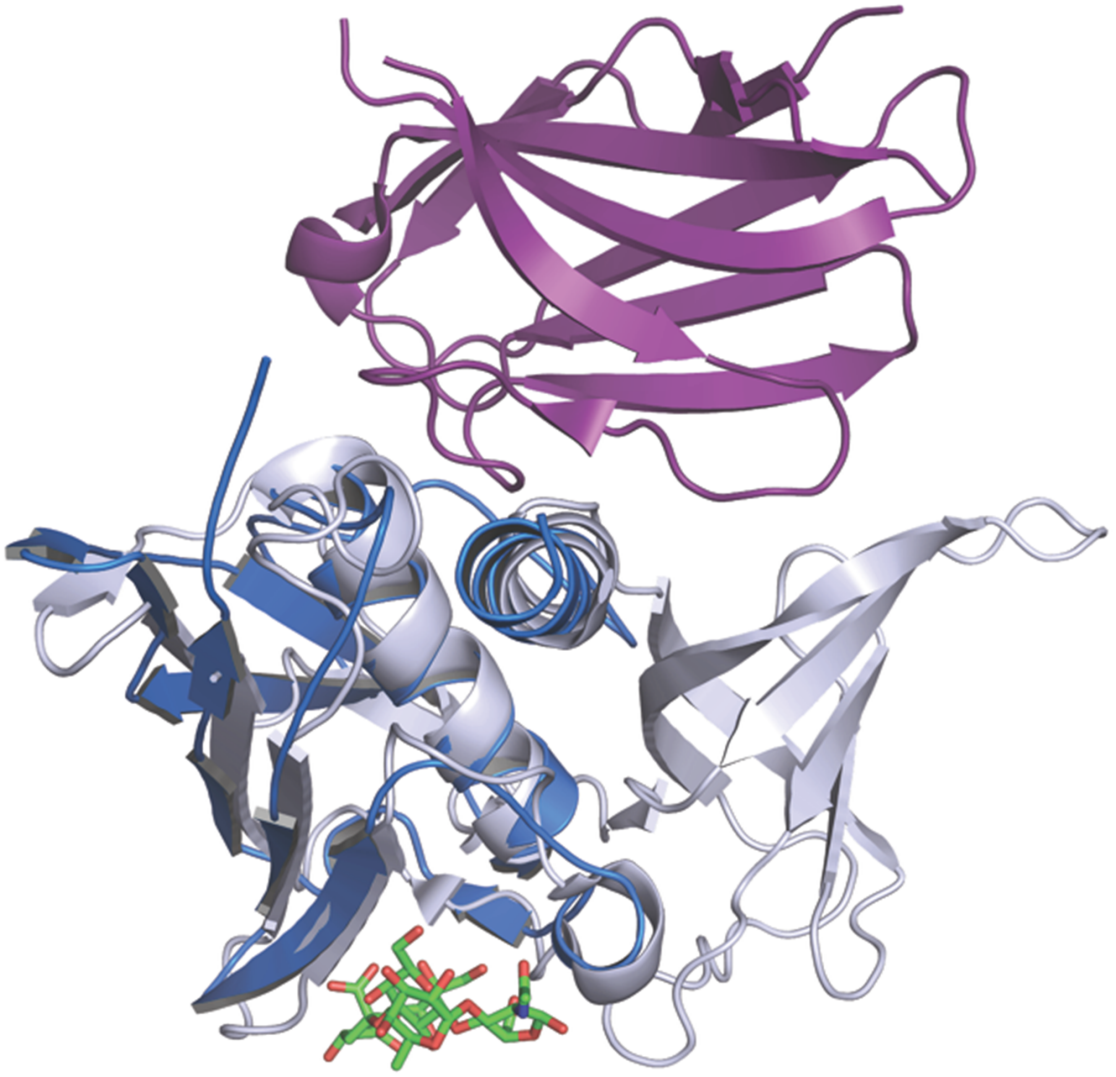
Comparison of SElX with TSST-1 in complex with human Vβ2. The structure of SElX (blue) with sLeX bound (in green) is overlaid with the structure of TSST-1 (silver) in complex with the Vβ2 region (purple) of a human TCR molecule (PDB: 2IJ0).

TSST-1 interacts with the TcRVβ chain via an interface that includes the back of the N-terminal α-helix, the central α-helix of the β-grasp domain, and the top of the OB-fold [44]. A structural overlay of SElX with the structure of TSST-1 in complex with TcRVβ2 reveals that this unique SAg has the potential to bind TcRVβ in a similar fashion to TSST-1. The β-grasps of both proteins overlay very well as do their N-terminal α-helices (Fig 6). This structural overlay places the TcR β-chain in close proximity to SElX. In particular, the N-terminal α-helix, the end of the central α-helix and the linker loop region that connects the N-terminus of SElX with the β-grasp domain (Fig 6). The overlay described here shows that SElX has the potential to bind TcRVβ predominantly via its spatially conserved N-terminal and central α-helices and potentially compensates for the lack of any OB-fold-TcRVβ interaction by the proximity of its connecting loop region to the TcRVβ chain. It is also evident from this structural overlay that the positioning of the potential TcRVβ binding site is on the opposite face of SElX to its sialylated glycan binding site and also leaves the concave face of β-grasp exposed for additional host interactions.

SAgs have two known binding sites for MHC class II. A hydrophobic ridge in the OB-fold domain allows for binding to the invariant MHC class II α-chain, while three conserved residues in the β-grasp domain participate in the tetravalent co-ordination of a zinc molecule with an invariant histidine on the polymorphic MHC class II β-chain [45–49]. Having no OB-fold domain means that SElX cannot interact with MHC class II via the traditional α-chain-binding site. Additionally, no structural homology to the zinc-co-ordinating residues is identifiable in the β-grasp of SElX.

### MHC class II binding is sialylated glycan-dependent but PBMC stimulation is not

**Fig 7:**
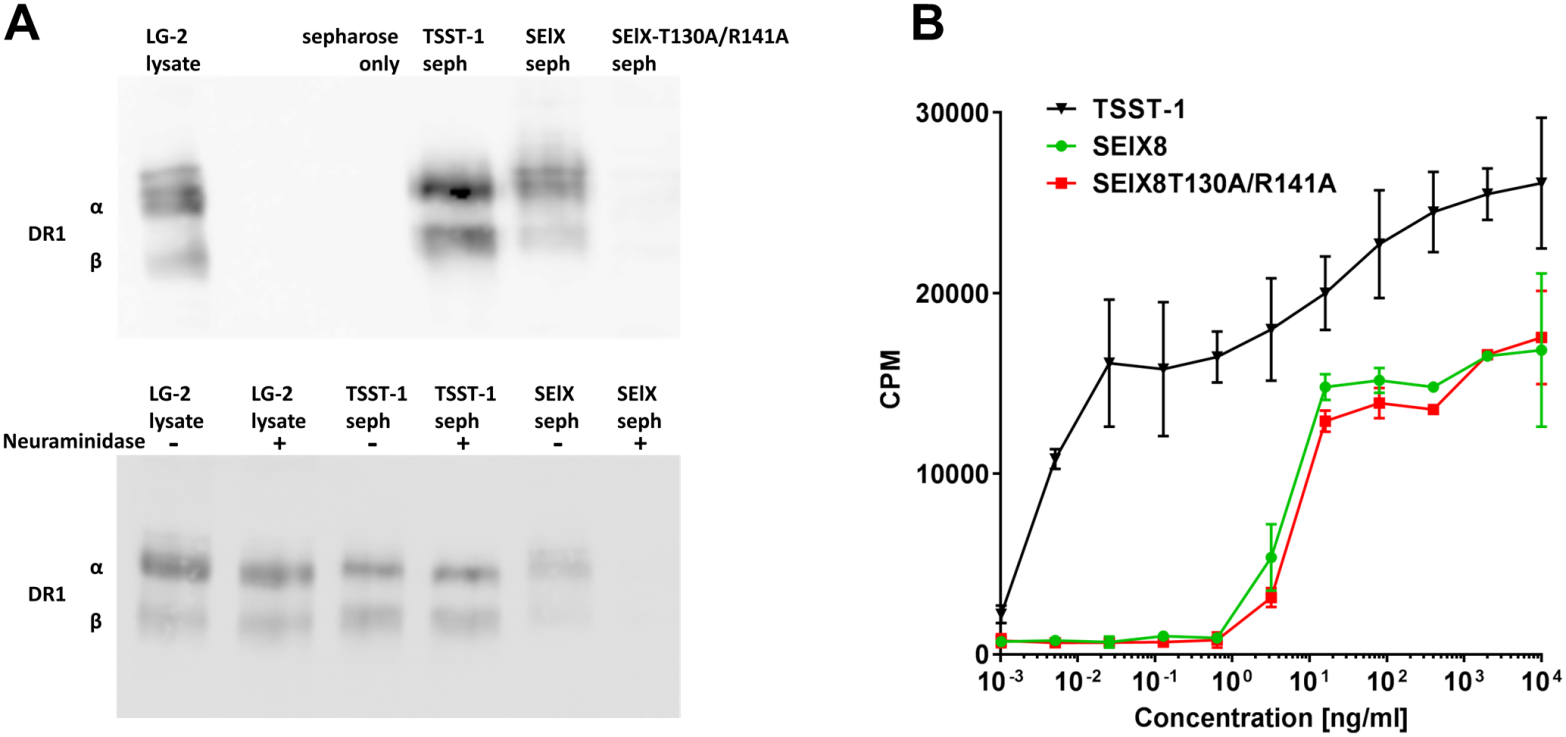
Association of the SElX glycan-binding site with MHC class II and T cell activation. (A) Binding of SElX to MHC class II. Upper panel—immunoassay detecting for DR1 isolated from LG-2 cell lysate by TSST-1, SElX, or SElX-T130A/R141A coupled to sepharose. Lower panel—immunoassay detecting for DR1 isolated from LG-2 cells lysates treated ± with neuraminidase by TSST-1 or SElX coupled to sepharose. LG-2 lysate is run as a control to indicate the α and β chains of DR1. Sepharose only is a control for non-specific binding. (B) Effect of the SElX glycan-binding site on superantigen activity. Proliferation of human PBMCs by SElX, SElX-T130A/R141A, and TSST-1 measured by the incorporation as counts per minute (cpm) of ^3^H-thymindine. The data (mean ± SD) is a representative of the PBMC assay which was performed in triplicate on cells isolated from at least three healthy individuals.

The ability of SElX to bind MHC class II was tested. SElX8 conjugated to sepharose was used to isolate MHC class II from cell lysates of the MHC class II (HLA-DR1 allele)-expressing cell line LG-2 [50]. Immunoblot analysis using a polyclonal antibody against HLA-DR1 showed SElX isolated bands consistent with those of the MHC class II alpha and beta chains recognised by the anti-DR1 antibody (Fig 7A). MHC class II was also affinity isolated by TSST-1 conjugated to sepharose but negligibly isolated by SElX8-T130A/R141A. This confirms that there is a sialylated-glycan-dependent binding of SElX to MHC class II, rather than the traditionally identified SAg binding sites. To support this, TSST-1 bound MHC class II from both neuraminidase-treated and untreated LG-2 cells, whereas SElX only isolated MHC class II from untreated cells. Glycan binding did not appear to influence the superantigen activity of SElX however, since both SElX and SElX-T130A/R141A displayed an equivalent capacity to stimulate the proliferation of PBMCs (Fig 7B). Both SElX and its glycan-binding site mutant displayed ½ maximal PBMC stimulation at approximately 10ng/ml, whereas TSST-1 had a more typical superantigenic potential with its ½ maximal stimulation in the low pg/ml range [21].

### Protection of *S*. *aureus* by SElX in a whole blood killing model of bacteraemia is glycan-binding dependent

**Fig 8:**
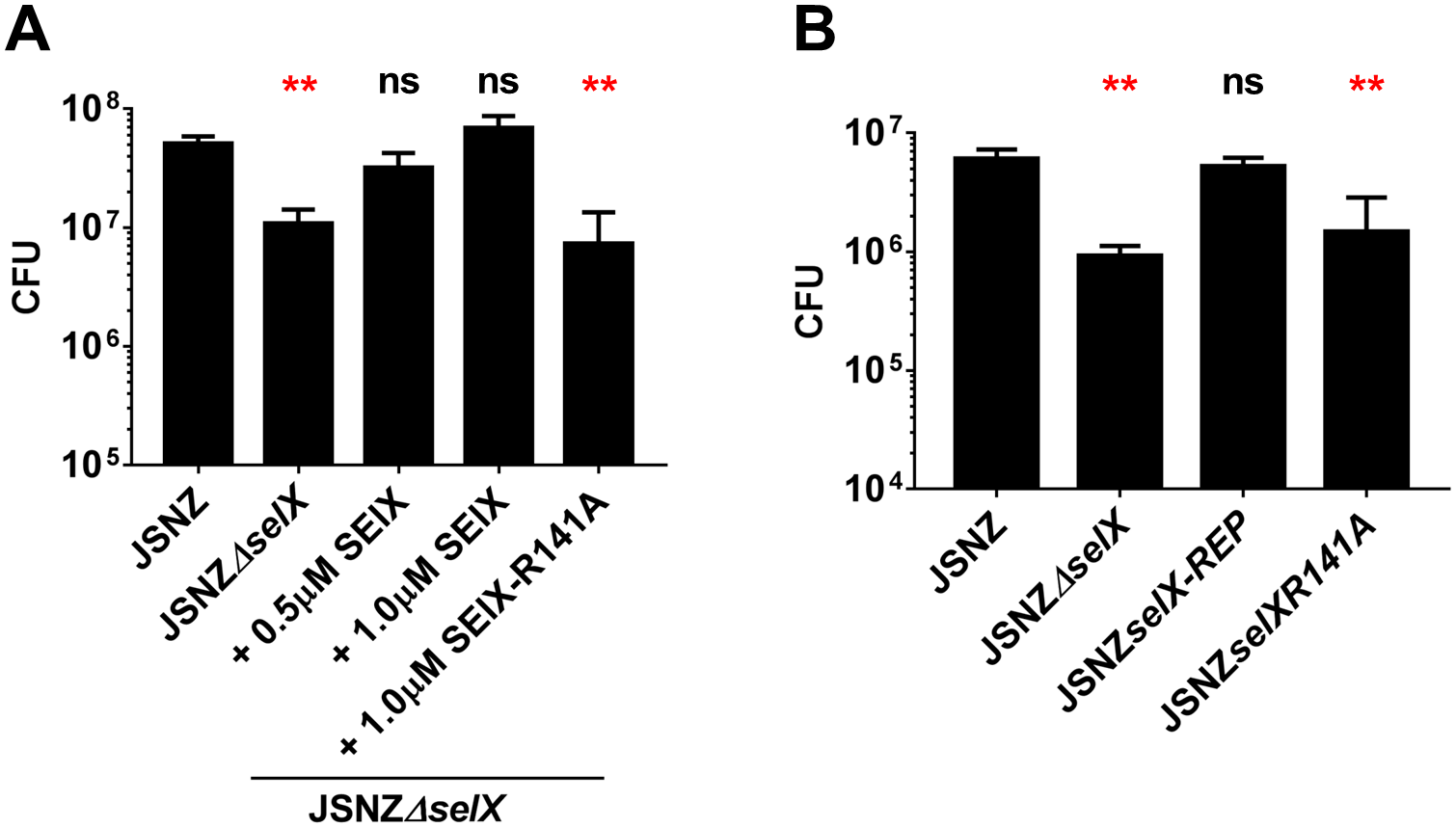
Contribution of SElX to *S*. *aureus* survival in whole blood. (A) Whole blood killing (WBK) of *S*. *aureus* strain JSNZ or JSNZ*ΔselX* +/- recombinant SElX (0.5 or 1.0 μM) or SElX-R141A at 1.0 μM. Bacterial survival was determined by CFU enumeration after 20 hr co-incubation. (B) WBK of *S*. *aureus* JSNZ, JSNZ*ΔselX*, JSNZ*selxR141A*, or JSNZ*ΔselxREP*. Bacterial survival was determined by CFU enumeration after 20 hr co-incubation. The graphs are a representative of three independent experiments performed on three individual donors. Data is the mean ± SD of duplicate tests enumerated in triplicate. Statistics were performed using Graphpad Prism. Kruskal—Wallis one way ANOVA was performed (p = 0.0005) and comparisons between samples and JSNZ were made using Dunn's Multiple Comparison Test (** p< 0.01, ns = not significant).

The contribution of SElX to the survival of *S*. *aureus* was studied using a whole blood killing assay designed to represent an *in vitro* model of bacteraemia. Deletion of *selX* from strain JSNZ (*JSNZΔselX)* resulted in a significant reduction in its ability to survive in human blood (Fig 8A). Addition of recombinant SElX to the assay increased *JSNZΔselX* survival. Complementation with the glycan-binding site mutant SElX-R141A did not increase the survival of *JSNZΔselX* in human blood indicating that the protective effect of SEIX was dependent on the sialylated glycan-binding site. To further validate this, *S*. *aureus* JSNZ strains were generated in which the deleted *selX* gene was replaced with either a glycan-binding site mutant gene *selXR141 (JSNZselXR141A)*, or repaired by the re-introduction of *selX* (*JSNZselX-REP*). Survival of the *selX* repaired strain, *JSNZselX-REP*, was comparable to wild type with significantly higher cfu’s recovered from both of these strains compared to JSNZ*ΔselX*. The mutant of *S*. *aureus* carrying the glycan-binding site mutated *selX* gene, *JSNZselXR141A*, showed a significant reduction in survival compared to wild type and did not provide any significant protective advantage over the *selX* deletion mutant from which it was derived (Fig 8B). All the modified strains displayed comparable *in vitro* growth curves and immunoblot analysis confirmed that *JSNZselX-REP* and *JSNZselXR141A* both produced SElX (S4 Fig). A comparison between *S*. *aureus* JSNZ and JSNZ*ΔselX* was made using mouse models of subcutaneous infection and systemic infection. No significant difference in survival was observed between the wild type bacteria and the SElX-deficient strain in either model (S5 Fig).

### Expression and regulation of *selX*

**Fig 9:**
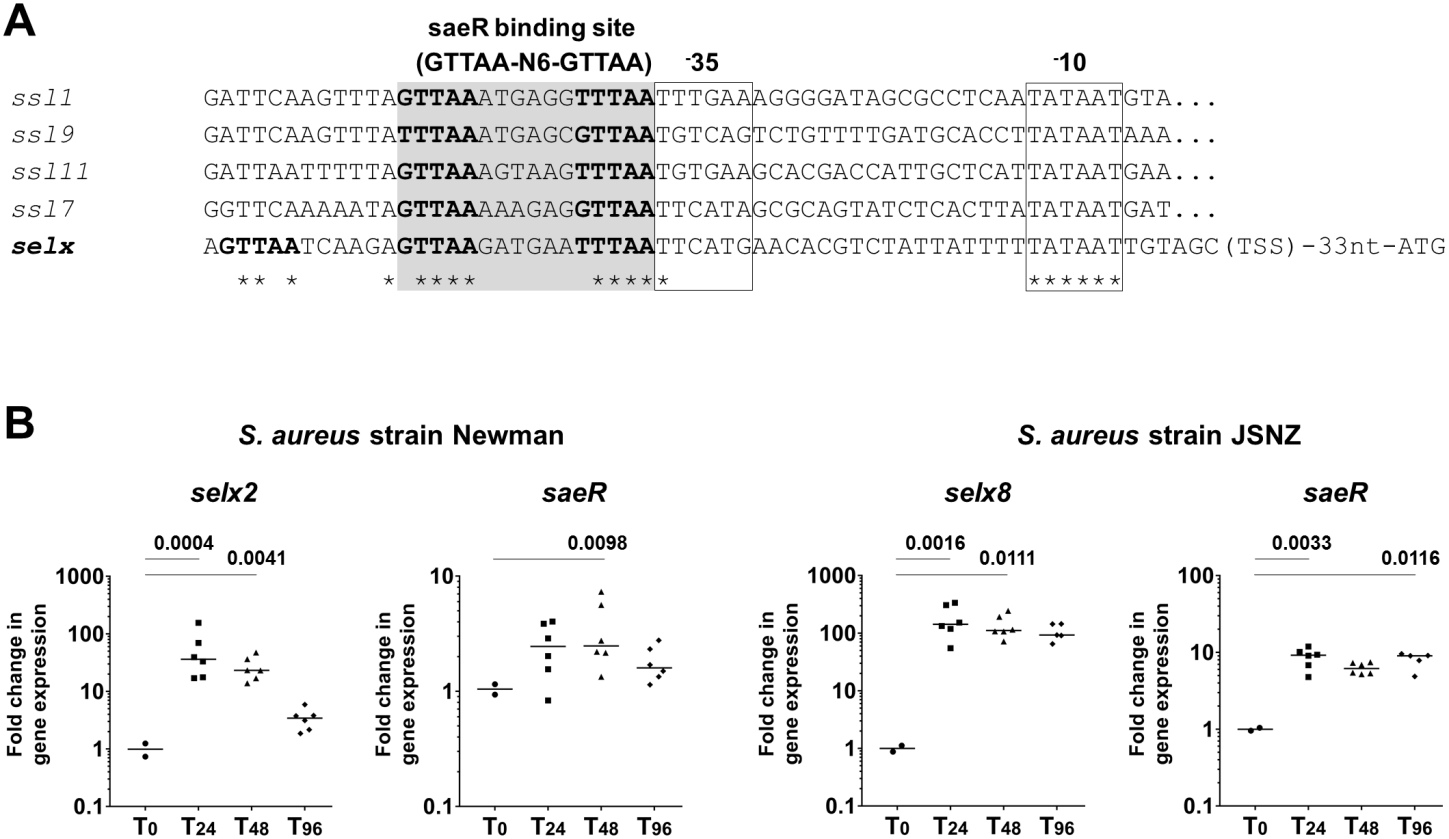
Expression analysis of *selX*. (A) Alignment of the region upstream from *selX* with the known upstream regulatory regions of *ssl1*, *ssl7*, *ssl9*, and *ssl11*. The -10 and -35 promoter elements are boxed and the region of homology to the saeR binding site (GTTAA-n6-GTTAA) is highlighted in grey. Conserved residues are indicated with an asterisk. (B). Relative *selX* transcript levels in *S*. *aureus* isolated from abscesses (n = 6) removed at 24 hr, 48 hr, or 96 hr after subcutaneous infection of mice with 5x10^6^ *S*. *aureus* Newman or JSNZ. *selx*, *saeR* expression normalized to reference genes *gyrB* and *ftsZ* and compared to *in vitro* expression of the inoculum. Each data point is the mean of triplicate values from an individual abscess, with the median of these mean values shown. Statistics were performed using Graphpad Prism. Kruskal—Wallis one way ANOVA was performed and comparisons between the *in vivo* samples and the inoculum were made using Dunn's Multiple Comparison Test. The p value of significantly different groups is shown.

Analysis of the 5’ untranslated region of *selx* was conducted to look for regulators of *selx* expression. A recent study into the control of *ssl1*, *ssl7*, *ssl9*, and *ssl11* expression revealed promoter elements that include a binding site for SaeR, the response regulator of the *S*. *aureus* exoprotein expression (Sae) two component system (TCS) 5’ to the translational start site of these genes [51]. Comparison of the upstream regions of *selx2* and *selx8* with those of these *ssls* confirmed that the *selx* gene also possesses a direct repeat sequence with high identity to the conserved SaeR binding site [52, 53]. This sequence, GTTAA(n_6_)GTTAA is seen directly upstream from the -35 and -10 promoter elements. An additional ½ SaeR binding site was identified 6 bases further upstream. By performing 5’ RACE the transcription start site for *selx* was identified 7 bases 3’ to the entirely conserved Pribnow box (or -10 promoter sequence) (Fig 9A).

Using a mouse model of subcutaneous infection, the expression levels of *selx* transcripts present in *S*. *aureus* strain Newman and JSNZ abscesses at 24, 48, and 96 hours after infection were compared with that of *in vitro* culture of the inoculum. During *S*. *aureus* Newman subcutaneous infection, an up-regulation of expression was observed from the earliest time point that was approximately 30-fold greater than the *in vitro* culture at the time of infection (Fig 9B). A corresponding trend towards increased *saeR* transcript expression was also observed at day one that declined in conjunction with that of *selx* expression. Expression of *selx* during subcutaneous infection of mice with *S*. *aureus* JSNZ was increased over 100-fold at 24 hrs in relation to the inoculum and remained elevated over the four day study. A robust and sustained increase in *saeR* was also measured over this time period (Fig 9B). In support of *selx* regulation by Sae, immunoblot analysis of the culture supernatant from an Sae TCS-deficient strain of *S*. *aureus* revealed the complete absence of any SElX production (S4 Fig).

## Discussion

The functional and structural insights gained from this research reveal that SElX is a unique member of the SAg family that shares features of the related SSL family. Though it was previously described as a SAg [41], we have shown that it also possesses the conserved sialylated-glycan binding site of the SSLs. It shows similar host factor binding profiles to the SSLs and mutation of key conserved residues in the binding site greatly reduces this toxins binding capacity for host proteins. The specificity of SElX, as determined by glycan array screening, was comparable to that of the SSLs, with SPR analysis confirming its affinity for sLacNac and sLeX. The affinity of SElX for sLacNac (14 μM for SElX8 and 23 μM for SElX2) is lower than the SSLs which range from 0.47 μM for SSL4 to 2.4 μM for SSL11 [42]. Perhaps this is a consequence of it lacking a glycan-binding aspartic acid that is conserved in the SSLs. SElX possesses a valine at this position that does not interact with sLeX in the crystal structure. The co-crystallization of SElX with sLeX did show however that all the remaining residues that interact with the tetrasaccharide are conserved with those of the SSLs.

Many of the leukocyte proteins bound by SElX were adhesion molecules including P-selectin, PECAM1 and notably several integrin’s that have important roles in immune recognition and cell activation. It is evident from the annotation of protein function (S1 Table) that several of the proteins interacting with SElX are involved in coagulation. By binding to these molecules, SElX has the capacity to interfere with important host immune and wound healing functions during infection to help the bacteria evade destruction. When comparing the proteins identified by affinity interaction with SElX-T130A/R141A to those bound by SElX, it is clear that the interaction with cell surface receptors has been lost, with the exception of integrin alpha IIb and beta 3. The top scoring identified proteins bound by this glycan-binding site mutant are predominantly intracellular and cytoskeletally-related. It is not possible to determine from our approach which of these host proteins bind directly or indirectly to SElX so it is conceivable that some of these proteins are isolated in complex with glycoproteins that directly bind to SEIX. SElX may interact with cytoskeletal components or they may be present as a consequence of their connection with directly bound surface receptors. If SElX does bind cytoskeletal structures this would give it the potential to interfere with cellular rearrangements that affect cell movement, phagocytosis, receptor recycling, and even the release of granule contents. SElX may possess both glycan-dependent and -independent binding sites. The affinity precipitation assays showed that the glycan-site mutants retained some protein binding capacity. These targets may be intracellular proteins based on the mass spectrometry data and the observation that the glycan-binding site mutant of SElX displayed negligible cell surface binding by flow cytometry. The nature of these SElX-host interactions is a focus of further investigation.

We used the WBK assay as an *in vitro* representation of staphylococcal bacteraemia and the relevance of using whole blood for identifying correlates of *S*. *aureus* virulence has been highlighted recently [54–56]. This assay revealed that SElX was necessary and sufficient for successful survival of *S*. *aureus* in human blood. Removal of *selX* caused a reduction in bacterial load of almost 1-log (or 85%) that could be fully restored by the addition of SElX or the reinstatement of *selX*. What’s more, the protection afforded by SElX was determined to be reliant on its glycan-binding site and reveals a unique property that is more in keeping with immune evasion by the glycan-binding SSLs than superantigenic immunomodulation.

We found the consensus direct-repeat binding sequence for SaeR, the response regulator of the saeRS two-component regulatory system, in the immediate region upstream of *selx*. In support of this regulator being involved in the control of *selx* expression, we found there to be a complete lack of SElX produced by a Sae TCS-deficient mutant of *S*. *aureus*. This indicates that, like the SSLs, expression of SElX would be turned on in situations when the bacterium comes under stress such as from attack by host defence mechanisms [57–60]. SElX would likely be produced at the same early time point as the SSLs to perform complimentary functions in immune evasion. The protection provided by SElX to the survival of *S*. *aureus* in the whole blood killing assay supports its role in blocking host innate defences. The sustained expression of *selx* over the 96 hour course of murine subcutaneous infection seen in *S aureus* JSNZ may be a consequence of the more robust *in vivo* expression of *saeR* in this strain compared to that observed from Newman. It should be noted however that the Sae TCS is constitutively active in Newman [58, 61] and that could explain the lower relative increase in *selx* up-regulation *in vivo*. Indeed we observed that Newman produces more SElX *in vitro* than JSNZ (S4 Fig).

Despite the ability to bind mouse cellular and serum proteins, and up-regulation during murine subcutaneous infection, we did not see a significant effect of SElX when comparing the *selX* gene deletion mutant with wild type *S*. *aureus* in the murine models of infection. One reason for this may be the weaker binding capacity of SElX for mouse cells. It is also possible that the sialylation of receptors in the mouse may be sufficiently different in pattern and/or magnitude to preclude SElX from interacting with the same set of glycoproteins that it does with the human host [62, 63]. It has previously been reported that SElX does not contribute to disease severity in a murine model of pneumonia [64]. However, by deleting *selX* in *S*. *aureus* an attenuation of virulence has been observed in both a rabbit model of necrotizing pneumonia [41] and a bovine model of mastitis [65] suggesting that alternate species to mice are more appropriate for investigating the contribution of SElX during disease.

X-ray crystallography revealed the unique single-domain structure of SElX. It completely lacks an OB-fold domain and its spatially conserved N-terminal α-helix is linked to the β-grasp domain by a short unstructured region. In addition to the SSLs, *S*. *aureus* produces other β-grasp domain exoproteins that are involved in immune evasion. These include the Chemotaxis Inhibitory Protein of *S*. *aureus* (CHIPS) and the Formyl Peptide Receptor-like 1 Inhibitory Protein (FLIPr) [25, 66]. Both of these immune evasion molecules display conservation of sequence and structure in the region of the glycan binding site [25].

Being a single-domain SAg means that it would not be possible for SElX to interact with TcRs in the same manner as most of the traditional SAgs that typically bind the TcRVβ chain via an interface involving the cleft between the N-terminal α‐helix and the top of the OB‐fold [67, 68]. Rather it has the potential to bind the TcR in a similar fashion to TSST-1 by using predominantly its two α-helices and its novel linker region, suggesting that SElX has maintained the minimal requirement at its N-terminus for engaging the TcR. However, because the first 21 residues are not defined in the structure, they cannot be precluded from providing additional contacts with the TcRVβ. Further investigation by co-crystallization with TcRVβ and mutagenesis will confirm this. SElX exhibits no conservation with residues of the SAgs involved in MHC class II binding. We found that the interaction of SElX with MHC class II was significantly influenced by its glycan-binding site. Yet this did not correlate with its ability to stimulate the proliferation of PBMCs since SElX-T130A/R141A displayed an equivalent activity to SElX. The superantigenic capacity of SElX however, was less potent than that of the typical SAgs [21]. These observations suggest a different mode of action for the T cell stimulation activity observed for SELX. This is currently under investigation.

SElX is present in most *S*. *aureus* and is considered to have been acquired by an ancestor of the *S*. *aureus* species [41]. We have shown here that SElX possesses functions of two major related families of staphylococcal virulence factors, although it is less potent than typical SAgs and has weaker affinity for sialylated glycans compared to the SSLs. Perhaps SElX represents the missing-link between the related SAgs and SSLs and is the descendent of an ancestral staphylococcal virulence factor that had the properties of both these present-day families. While subsequent duplications of this ancestor led to the functional specialization seen in the SAgs and SSLs, SElX instead evolved to retain the functional properties of the precursor protein, losing its OB-fold domain in the process. Consequently, SElX targets both the adaptive immune system as a SAg and innate immune defences as an SSL. It is therefore unsurprising that this ‘SSL-like SAg' has been almost universally retained by all *S*. *aureus*.

With the increasing seriousness of antimicrobial resistance associated with *S*. *aureus*, there is a need for alternate therapeutic interventions. The discovery of virulence factors like SElX that correlate with disease and the determination of their modes of action will allow for a more targeted approach to the development of anti-infectives that can be used to treat or prevent staphylococcal disease.

## Materials and methods

### Ethics statement

Blood was collected from healthy human volunteers who had given informed consent in writing in accordance with the University of Auckland Human Participants Ethics Committee (UAHPEC) guidelines. Animals were housed and cared for in accordance with The Animal Welfare Act (1999) and institutional guidelines provided by the University of Auckland Animal Ethics Committee, which reviewed and approved these experiments under application R847. The mice were shaved and inoculated with *S*. *aureus* under isoflurane anaesthesia. They were monitored daily for alterations in body weight and general health. Mice were euthanized by CO_2_ inhalation.

### Protein production

The gene for TSST-1 was cloned from *S*. *aureus* strain RC31187 and the various SElX and SSL6 genes and mutants from *S*. *aureus* strain Newman or *S*. *aureus* strain JSNZ using the primers listed in Table 3. Mutants were generated by overlap PCR using internal overlapping primers containing the mutation together with the external cloning primers. The genes were cloned into pET32a-3C, recombinant proteins were expressed in *Escherichia coli* AD494(DE3)pLysS as thioredoxin fusion proteins, and isolated by nickel affinity chromatography (Ni Sepharose 6 Fast Flow, GE Healthcare). The thioredoxin was cleaved off with 3C protease and the proteins were further purified using ion-exchange chromatography (SElX, SSL6—MonoS, SSL11, TSST-1—MonoQ. GE Healthcare). Production of SSL11 has been described previously [34, 42].

**Table 3:** Primers used in this study.

Primer name	Primer Sequence
selX-for	CGGGATCCTCAACACAAAATTCCTCAAGTG
selX-rev	GCGAATTCTCAAACTTGTTCAATGTCATTAAC
selXnm-T1301A-for	GTGGTAAATATGCATTAGAGTCGCATAAAG
selXnm-T1301A-rev	CTCTAATGCATATTTACCACCATCTTTTG
selxnm-R141A-for	CAAAAAGATGCGGAAAATGTAAAAATTAATACAG
selXnm-R141A-rev	CATTTTCCGCATCTTTTTGTAGCTCTTTATG
selXj-T130A-for	CGGTAAATATGCATTAGAGTCGCATAAAGAG
selXj-T130A-rev	CTCTAATGCATATTTACCGCCATTCTTTG
selXj-R141A-for	CAAAAGAATGCGGAAAATGTAGAAATTAATACTG
selXj-R141A-rev	CATTTTCCGCATTCTTTTGTAACTCTTTATGC
SSL6-for	CGGGATCCGCAGAATCAACTCAAGGTCAACAC
SSL6-rev	GGAATTCTTATTTATATTCTAGCTCAACATTAATTTC
SSL6-R181A-for	CCGCATGCCATGGGTGACACGATAG
SSL6-R181A-rev	CACCCATGGCATGCGGTTGTAGTTTTTTTG
TSST-1 for	CGCCCGGGTCTACAAACGATAATATAAAGG
TSST-1 rev	GCGAATTCTTAATTAATTTCTGCTTCTATAG
seIX qFOR	TTGGGTTTATTCAGAGAGACCT
selX qREV	GTTACCTTTAGGCAAATGTTCTC
saeR qFOR	CCAAGGGAACTCGTTTTACG
saeR qREV	ACGCATAGGGACTTCGTGAC
gyrB qFOR	AAATCGCCTGCGTTCTAGAG
gyrB qREV	CCAGGTAAATTAGCCGATTGC
ftsZ qFOR	GGCGAGTCATTGTCATTA
ftsZ qREV	AATCCAGTGCTACCAGAT
selX-upper-for	CTAGATCGATGTCTTTTTTCAGTTATCCAATT
selX-upper-rev	ATTTAATTACCTCCTTGATGTA
selX-lower-for	TTTACATCAAGGAGGTAATTAAATGGCGGTAAATATACATTAGAG
selX-lower-rev	CTAGGAGCTCAAGATCACCTCTGACAAAATAT
selX-outer-for	CTTATCATTCCAAGCATAAG
selX-outer-rev	TAATGGGTTAAATTGATCTGTT
selX-REP-for	CATACACAGTCGCTGGCAGAGTGTATACACCTAAGAGG
selX-REP-rev	TCTGCCAGCGACTGTGTATG
selX-GSP1	CTCTTTATGCGACTCTAATG
selX-GSP2	TGACGATGTTACCTTTAGGCAAATGTTCTC
selX-GSP3	CTGCGAATTCTATTGTATCCTTGCTGTATC
Abridged anchor primer	GGCCACGCGTCGACTAGTACGGGIIGGGIIGGGIIG
Abridged universal amplification primer	GGCCACGCGTCGACTAGTAC

### Coupling of recombinant proteins to sepharose

Recombinant proteins were coupled to sepharose in accordance with the manufacturer’s guidelines. Protein at 2 mg/ml in PBS pH 8.0 was added to cyanogen bromide activated sepharose (GE Healthcare) to a final concentration of approximately 5mg protein/ml sepharose. The slurry was incubated with slow inversion at room temperature until a negligible amount of protein remained in the supernatant. Any remaining active sites on the sepharose were quenched by incubation in 100mM Tris pH8.0/150mM NaCl for 2 hours at room temperature before the sepharose was repeatedly washed with PBS pH 8.0/0.1% sodium azide and stored at 4°C as a 1:1 slurry in PBS/azide.

### Binding assays

Fresh human blood was collected in Heparin vacutainer tubes (BD Biosciences). Granulocytes, mononuclear cells and plasma were isolated by separation through a Histopaque 1077 over Histopaque 1119 (Sigma) double density centrifugation gradient according to the manufacturer’s instructions. LG-2 cells were prepared by culturing in complete RPMI-1640/10%FCS (Gibco). For neuraminidase treatment, 1x10^7^ LG-2 cells/mL in 150 mM NaCl, 5 mM CaCl_2_ pH 6.0 were incubated with or without 25 unit/ml neuraminidase (New England Biolabs) for 1 h at 37° in a 5% CO_2_ incubator with occasional mixing. To isolate mouse leukocytes, female BALB/c mice aged 5–6 weeks were culled via CO_2_ asphyxiation and the spleens, tibiae and fibulae were removed. Single cell suspensions of splenocytes were created by pushing the tissue through a sterile metal sieve. Any remaining cells still present on the sieve were washed through with sterile PBS. Bone marrow was flushed from cut bones with PBS using a 20-gauge needle to create single cell suspensions which were filtered through a 70 μm strainer. Red blood cells were removed by passing the cell suspensions through a Histopaque 1083 (Sigma) gradient. The cells were then washed with PBS. Cell lysates were prepared by incubating 1x10^7^ cells/ml in 10 mM Tris-HCl (pH 8.0), 140 mM NaCl, 1% (v/v) Triton X-100, 1 mM iodoacetic acid, 1 mM PMSF, 0.025% NaN_3_ for 1 hr at 4°C, before centrifugation at 20000g for 30 min. 10 μl of protein:sepharose slurry was added to 100 μl of cell lysate or plasma in a total volume of 0.5 ml 10 mM Tris-HCl (pH 8.0), 140 mM NaCl, 1 mM PMSF, 0.025% NaN_3_ and incubated with slow inversion for 30 min at room temperature. The protein:sepharose was washed three times in 10 mM Tris-HCl (pH 8.0), 140 mM NaCl, 1% TX-100, 1 mM PMSF, 0.025% NaN_3_ before being boiled in 2X sample buffer. The sample was separated by SDS-PAGE and visualized by Coomassie Blue staining or transferred to nitrocellulose membranes for Immunoblot analysis. Membranes were blocked at 4°C O/N in 10 mM Tris (pH 8.0), 120 mM NaCl, 0.1% Tween-20, 5% Non-fat milk powder, probed for 1 hour at room temperature with rabbit anti-SElX polyclonal IgG (made in-house) or rabbit anti-huDR1 polyclonal IgG (made in-house), followed by 1 hr with goat anti-rabbit IgG-HRP (Dako). Chemiluminescence was performed using SuperSignal West Pico Chemiluminescent Substrate (Thermo Scientific) and images were captured using a LAS-3000 imager with Image Reader software (Fujifilm).

### Identification of the host proteins bound by SElX using mass spectroscopy

Heparinised blood was incubated in erythrocyte lysis buffer (150 mM NH_4_Cl, 10 mM KHCO_3_, 0.1 mM EDTA, pH 7.4) and washed 3 times in PBS. The isolated leukocyte fraction was lysed at 1x10^7^ cells/ml in 10mM Tris-HCl (pH 8.0), 140mM NaCl, 1% (v/v) Triton X-100, 1mM iodoacetic acid, 1mM PMSF, 0.025% NaN_3_ for 1hr at 4°C prior to clarification by centrifugation at 20000xg for 30 min. 10μl of protein:sepharose slurry was added to 100μl of cell lysate in a total volume of 0.5ml 10mM Tris-HCl (pH 8.0), 140mM NaCl, 1mM PMSF, 0.025% NaN_3_ and incubated with slow inversion for 30 min at room temperature. The protein:sepharose was washed three times in 10mM Tris-HCl (pH 8.0), 140mM NaCl, 1% TX-100, 1mM PMSF, 0.025% NaN_3_. The protein:sepharose samples were incubated in 50μl elution buffer (6M urea/2M thiourea, 20mM tris (pH 8.0), 20mM NaCl, 5mM DTT) for 30 min at room temperature with frequent mixing. The samples were centrifuged and the supernatant removed with a Hamilton syringe. A further 20 μl of elution buffer was added to the beads and incubated for 10 min. The supernatants from the two elutions were combined and centrifuged again. 50 μl was taken out, snap frozen in ethanol-dry ice and stored at -80C for mass spectroscopy analysis. 10 μl of the remainder was mixed with 10 μl 2x sample buffer and 10μl of this was analysed by SDS-PAGE. Protein identification was performed by LC-MS/MS using a Sciex TripleTOF 6600 by the Mass Spectrometry Centre, Auckland Science Analytical Services, The University of Auckland, Auckland, New Zealand. The data was analysed using ProteinPilot 5.0.

### Alexa Fluor 488 labelling of SElX

Recombinant SElX or SElX-T130A/R141A were coupled to Alexa Fluor 488 based on the manufacturer’s recommendations. One twentieth volume of 10 mg/ml Alexa Fluor 488 (Life Technologies) in DMSO was added to 10 mg/ml SElX in 0.1 M NaHCO_3_ pH 8.3 and incubated in the dark at room temperature for 2 hours. SElX conjugated with Alexa Fluor dye (SElX-488 and SElX-T130A/R141A-488) were separated from free label using a HiTrap Desalting column (GE Healthcare) in PBS pH7.4.

### Flow cytometry

Heparinised whole human blood was incubated in erythrocyte lysis buffer. The remaining leukocytes were washed in PBS and suspended at 1 x10^7^ cells/ml in FACs buffer (PBS/1% BSA). A two-fold dilution series of SElX-488 from 500nM was incubated with 1 x10^6^ cells at room temperature for 15 min. For competition assays, 100 nM SElX-488 was added to 1 x10^6^ cells with or without addition of 100, 500, or 1000 nM unlabelled SElX or SElX-T130A/R141A and incubated at room temperature for 15 min. Mouse cells were processed as described above. 1x10^6^ mouse cells +/- 100nM SElX-488 were incubated in FACs buffer for 15 min with appropriate antibodies to identify the following populations: CD3-PE-Cy5 to identify T cells in the spleen; B220-Cytochrome to identify B cells in the spleen and bone marrow; and Gr-1-APC-Cy7 to identify myeloid cells, predominantly neutrophils, in the bone marrow. Cells were washed in FACs buffer prior to acquisition using a BD LSR II Flow Cytometer with FACsDiva (BD Biosciences). Data analysis was performed using FlowJo (FlowJo, LLC) with cell populations gated as granulocytes, monocytes, and lymphocytes based on size and granularity, or by cell-specific marker expression. Analysis of human leukocyte binding was performed in triplicate using 3 healthy individuals. The mouse cell binding was performed twice using n = 1 mouse each repeat. Statistical analysis was performed using Graphpad Prism.

### Live cell imaging

Live cell imaging was performed as previously described [42]. Briefly, 1×10^5^ neutrophils were adhered to L-lysine coated glass bottom dishes (World Precision Instruments) for 30 min at RT in PBS pH 7.4. 0.2 μM SElX-488 was incubated with the cells for 15 min at either 4°C or 37°C in PBS pH 7.4. Excess SElX-488 was washed away with PBS pH 7.4 before being viewed by the Olympus FV1000 confocal scanning microscope at 600× magnification. The analysis software used was Olympus Fluoview v1.7b with resizing performed by ImageJ v1.46.

### SElX glycan binding specificity by glycan array screening

SElX-488 was sent to the Consortium for Functional Glycomics (CFG): Protein-Glycan Interaction Core (http://www.functionalglycomics.org/static/consortium/resources/resourcecoreh.shtml) for screening of their mammalian glycan array. Binding was analysed at 100, 200, and 500 μg/ml to version 5.0 of the printed array consisting of 611 glycans in replicates of 6. Relative binding was measured as relative fluorescent units (RFU). The average RFU value from the replicates, the standard deviation, and %CV (%CV = 100 X Std. Dev / Mean) were calculated after removing the highest and lowest values from each set of 6. The SElX-488 used for glycan screening is referred to as SSL0 on the consortium website and data from the screening can be found via the following link: (http://www.functionalglycomics.org/glycomics/search/jsp/result.jsp?query=ssl0&cat=all).

### Surface plasmon resonance (SPR) analysis

Biosensor analysis of SElX interactions with sLeX and sLacNac were performed on a Biacore T200 (GE Healthcare, Uppsala, Sweden). Ligands were coupled using carbodiimide chemistry to a CM5 biosensor chip surface according to manufacturer’s instructions. BSA-sLeX and BSA-sLacNac (Dextra Laboratories) were coupled at 200–250 RU in 100 mM Na-Formate pH4.3. Remaining sites were blocked with BSA. Control channels for subtraction of bulk and non-specific responses were coupled with BSA to similar levels as test channels. SElX2 and the trailing edge from size exclusion chromatography of SElX8 (concentration series from 50–0.25 μM), in HBS-EP^+^ (0.01 M HEPES pH7.4, 0.15 M NaCl, 3 mM EDTA, 0.05% Surfactant P20, GE Healthcare, Uppsala, Sweden), were passed over the immobilised ligands at 30 μl/min. The response at equilibrium (Req) was measured as the binding response plateau at 5 min. Surfaces were regenerated between cycles with 4 M GuCl. Equilibrium binding data were fitted to a steady state single binding site model using the Biacore T200 Evaluation software (GE Healthcare). For comparison with binding site mutants, SElX proteins (20 μM) were passed over the immobilised ligand at 30 μl/min for 100 s. Sensorgram overlays were carried out using the Biacore T200 Evaluation software (GE Healthcare). Each experiment was performed in duplicate and repeated three times. The affinity (K_*D*_) values are expressed as mean ±SD of the repeats.

### Proliferation assays

Proliferation assays were performed in 96 well U bottomed plates. Peripheral Blood Mononuclear Cells (PBMCs) isolated by Histopaque 1077 (Sigma) density centrifugation were suspended at 1x10^6^ cells/ml in complete RPMI-1640/10% FCS (Gibco) and then added in an equal volume to a 10-fold dilution series (in triplicate) of toxin starting from 20μg/ml in complete RPMI-1640/10% FCS (Gibco). The plates were incubated at 37°C in 5% CO_2_ for 3 days. 0.25 μCi ^3^H-thymidine was added to each well and the plates incubated for a further 18hr. The plates were harvested to filter mats and incorporation of ^3^H-thymidine into cellular DNA was determined using a Wallac Jet 1450 Microbeta Trilux liquid scintillation counter (Wallac).

### Generation of *selX* deletion, *selXR141A*, and *selX* repaired strains of *S*. *aureus* JSNZ

To generate *JSNZΔselx* the flanking regions of *selx* were amplified by PCR from JSNZ genomic DNA using the primers selX-upper-for with selX-upper-rev, and selX-lower-for with selX-lower-rev (Table 3). The upper and lower flanking region PCR products were then mixed and used as template for PCR with the selX-upper-for and selX-lower-rev primers. The resulting product was cleaved at the primer-introduced restriction sites, ligated with pIMAY cleaved with the same endonucleases, and transformed into *E*. *coli* DC10B. After sequence confirmation the plasmid isolated from DC10B was electroporated into JSNZ. Integration of pIMAY into JSNZ and excision of *selX* was performed as described by Monk *et*. *al*. 2012 [69]. Confirmation of gene deletion was confirmed by sequencing with the selX-outer-for and selX-outer-rev primers (Table 3). To generate *JSNZselxR141A*, JSNZ genomic DNA was amplified using selX-upper-for primer with the selXj-R141A-rev primer, and selX-lower-rev primer with the selXj-R141A-for primer (Table 3). The two PCR products were mixed and used as template for amplification using the selX-upper-for and selX-lower-rev primers. To generate *JSNZΔselx-REP*, JSNZ genomic DNA was amplified using selX-upper-for primer with the selX-REP-rev primer, and selX-lower-rev primer with the selX-REP-for primer (Table 3). The overlapping REP primers were designed to introduce a single synonymous substitution into the *selx* gene [41]. The two PCR products were mixed and used as template for amplification using the selX-upper-for and selX-lower-rev primers (Table 3). The resulting products were introduced into pIMAY using the primer-introduced restriction sites and transformed into DC10B. Following sequence confirmation each plasmid was electroporated into *JSNZΔselx*, allelic exchange was performed as described [69], and introduction of *selXR141A* or *selX-REP* was confirmed by sequencing.

### Whole blood killing assay

Overnight cultures of *S*. *aureus* JSNZ, JSNZ*Δselx*, JSNZ*selxR141A*, or JSNZ*selx-REP* in tryptic soy broth were diluted 1/100 and cultured at 37°C until mid log-phase. After suspension in Hanks balanced salt solution to an OD600 of 0.4 (= ~1x10^8^ cells/ml), 1x10^5^ CFU *S*. *aureus*, JSNZ*Δselx*, JSNZ*selxR141A*, or JSNZ*selx-REP* were incubated with 70% whole blood with or without recombinant SElX at the indicated concentrations for 20 hr at 37°C with gentle shaking. Dilutions of the suspensions at time 0 and after 20 hr were plated in triplicate onto tryptic soy agar and incubated O/N at 37°C for enumeration. Each assay was performed in duplicate with enumerations made in triplicate on at least three individual donors. Statistics were performed using Graphpad Prism. Kruskal—Wallis one way analysis of variance (ANOVA) was performed and comparisons between samples were made using Dunn's Multiple Comparison Test.

### Transcriptional analysis of *selX* during murine subcutaneous infection

Subcutaneous infection of mice with *S*. *aureus* Newman or JSNZ was performed as previously described [43]. Log-phase *S*. *aureus* were washed and diluted in PBS and then mixed 1:1 in a sterile cytodex bead (Sigma) solution (0.5 g/ml in PBS). Female CD1 mice aged 7–8 weeks were anaesthetized with isoflurane, the flank area shaved and 5x10^6^ CFU bacteria was injected subcutaneously into the flank. Mice were euthanized by CO_2_ inhalation and abscess tissue (from 2 independent experiments containing n = 3 mice per treatment group) was aseptically collected from groups of mice at 24, 48, and 96 hours post infection. For transcript analysis, 1 ml RNAprotect Bacteria reagent (Qiagen) was added to each abscess immediately upon excision. The samples were pelleted, suspended in a further 1 ml of RNAprotect Bacteria reagent and pelleted again. The pellets were suspended in 0.3 ml TE buffer containing 25 μg lysostaphin (Sigma) and incubated for 1 hr. Seven 0.1 mm silica/zirconia beads (Omni) and 1ml Trizol LS reagent (LifeTech) were added and the samples beaten in an Omni BeadRupter-24. After addition of chloroform the samples were centrifuged and the extracted RNA was ethanol precipitated. Contaminating DNA was removed using Turbo DNase (Ambion) and the bacterial RNA was enriched for using a Microbenrich kit (Ambion) and amplified using a MessageAmp II Bacteria kit (Ambion). RNA was extracted from the inoculum as for the abscess samples (with exclusion of the Microbenrich step) to provide for *in vitro* comparisons. Complementary DNA was synthesized from total RNA using Superscript lll first strand synthesis supermix (LifeTech) and stored at -80°C. Real time PCR analysis was performed in triplicate using an Applied Biosystems 7900HT Fast Real-Time PCR System with PerfeCTa SYBR Green FastMix ROX (Quanta Biosciences) on 1/2 dilutions of the synthesized cDNA and *selX*-specific primers (Table 3). Real time PCR data was normalised against the reference genes *ftsZ* and *gyrβ*. Arbitrary gene expression values were converted to ratios against an average of the *in vitro* data.

### Determination of the *selX* transcriptional start site

The identification of the *selX* transcriptional start site was achieved using the method described by Miller *at*. *al*. 2015 [70]. Briefly, first strand cDNA was synthesised from *S*. *aureus* RNA using selX-GSP1 (Table 3) and SuperScript RT III/RNAse First Strand Synthesis Mix (Life Technologies) according to the manufacturer’s instructions. After incubation at 70°C to terminate the reaction, the cDNA was incubated at 37°C with RNase H (Thermo Fisher Scientific), then column purified (Zymo Research). A poly-C tail was added to the 3’ end of the cDNA using Terminal Deoxynucleotidyl Transferase (Thermo Fisher Scientific) and then amplified using selX-GSP2 and the Abridged anchor primer (Table 3). A nested amplification round of PCR was performed on the product using selX-GSP3 and the Abridged universal amplification primer (Table 3). The nested product was isolated by agarose gel electrophoresis and extracted using a NucleoSpin Gel and PCR clean-up kit (Macherey-Nagel). The product was cloned into pBluescript using the restriction enzymes EcoRI and SalI and the plasmid from six positive transformants were sequenced.

### Crystallization of SEIX

SEIX (10 mg/ml) was co-crystallized with 5 mM sLeX (Dextra Laboratories) in the presence of 24% polyethylene glycol (PEG) 3350 and 250 mM Tri-Lithium citrate pH 7.5 at 17°C. Protein crystals formed within 7 days. The protein crystal was flash-cooled in the same crystallization condition supplemented with 20% glycerol. Diffraction data was collected at 1.5418 wavelength after 2x 1 sec room temperature annealing. The protein structure of SEIX8 in complex with sLeX was solved by molecular replacement with a partial model of SSL4 (PDB: 4DXG) residues 130–200 and was refined at 1.66 Å using Phaser MR and REFMAC in CCP4 suites (Table 2). Structural comparisons of the glycan binding sites were made using LSQ in coot for all atom rmsd.

The atomic coordinates and structure factors for SElX complexed with sLeX have been deposited in the PDB under code no. 5U75.

## Supporting information

S1 Table Mass spectrometry data. (XLSX)

S1 Fig Sequence comparison of SElX with the SAgs and SSLs. (A) Amino acid alignment of the Group A Streptococcal and Staphylococcal SAgs with the Staphylococcal Superantigen-Like (SSL) proteins in the region of the PROSITE signature sequence PS00278. The consensus sequences of the SSLs [KE(L/I)D] and the SAgs [QE(L/I/V)D] are highlighted in yellow. The Lysine (K) of this motif conserved in the SSLs and the Glutamine (Q) of the SAgs are shown in bold type. (B) Structural alignments of selected *S*. *aureus* SAgs and SSLs showing the MHC class II α-chain binding region (upper panel) and MHC class II β-chain binding region (lower panel) generated using PROMALS3D (PROfile Multiple Alignment with predicted Local Structures and 3D constraints) (http://prodata.swmed.edu). Secondary structural elements are shown below the alignments. Amino acids that have been experimentally determined to bind MHC class II are shown highlighted in blue (MHC class II α-chain binding) or green (MHC class II β-chain binding). Amino acids from SSLs that are involved in binding to sialylated glycans are highlighted in yellow. Those that have been determined to bind sLeX by X-ray crystallography are shown in bold type.
(TIF)

S2 Fig Host protein binding by SElX. (A) The binding profile comparison of SElX2 and SElX2-R141A. Proteins from cell lysates of human PMN, PBMC, platelets, or from human plasma pulled out of solution by binding to SElX2-sepharose, SElX2-R141A-sepharose, or sepharose alone, and separated by reducing and denaturing SDS-PAGE (12.5%) alongside the Benchmark Protein Marker (Life Technologies). (B) Energy- and sialylated-glycan-dependent binding of SElX to neutrophils. SElX2 conjugated to Alexa Fluor 488 (SElX-488) was incubated with human neutrophils and monitored by live-cell confocal microscopy. After 15 minutes of incubation at 37°C intense and localized intracellular staining of SElX2 similar to that previously described for SSL4 and SSL11 was observed whereas no internalization was seen at 4°C. No cell staining could be seen using fluorescently labelled SElX2-T130A.
(TIF)

S3 Fig Bar chart of the glycan binding profile of SElX to the functional glycomics consortium glycan array (version PA_v5). High affinity ligands bound by SElX are labelled with blue circles. The structures of the top binding glycans are shown in cartoon form. These are predominantly structures contain sialyl-lactosamine (sLacNac = Neu5Aca2-3Galb1-4GlcNAc) and sialyl Lewis X (sLeX = Neu5Acα2-3Galβ1-4(Fucα1–3)GlcNAc). N-Acetylneuraminic Acid (NeuAc) purple diamond, galactose (Gal) yellow circle, N-Acetylglucosamine (GlcNAc) blue square, fucose (Fuc) red triangle, and mannose (Man) green circle.
(TIF)

S4 Fig Comparison of the wildtype and *selx* mutants of *S*. *aureus* JSNZ. (A) Analysis of the *in vitro* growth of *S*. *aureus* Newman, Newman*ΔsaeQRS*, JSNZ, JSNZ*ΔselX*, JSNZ*selX-REP*, and JSNZ*selXR141A* at 37°C in tryptic soy broth. This data is a representative of two independent experiments and was performed in duplicate. (B) Detection of SElX production by Newman, Newman*ΔsaeQRS*, JSNZ, JSNZ*ΔselX*, JSNZ*selX-REP*, and JSNZ*selX-R141A*. A 5μl sample of culture supernatant from each of the indicated bacteria, growth O/N in RPMI, was, separated by SDS-PAGE (12.5%) alongside 1ng of rSElX2 and rSElX8 included as controls, under reducing and denaturing conditions, and transferred to nitrocellulose. Western analysis was conducted using affinity purified rabbit anti-SElX (made in-house) and the secondary antibody goat anti-rabbit IgG-HRP (AbD serotec).
(TIF)

S5 Fig Murine models of infection. (A) Subcutaneous infection of mice with JSNZ or JSNZ*ΔselX*. CD1 mice were injected subcutaneously on the left and right flank with 5 x 10^6^ CFU *S*. *aureus* JSNZ or JSNZ*ΔselX*. Tissue at the site of infection was removed and homogenised to estimate the CFU/abscess after 96 hours. Each treatment group contained n = 4 mice, each point represents a single abscess and the horizontal bar is the median value. There were no significant differences between the treatment groups (Mann-Whitney). (B) Intrperitineal infection of mice with JSNZ or JSNZ*ΔselX*. Mice were intraperitoneally injected with 1 x 10^8^ *S*. *aureus* JSNZ or JSNZ*ΔselX*. Spleen, Liver and Kidneys from individual mice were removed on day 5 post infection. Samples were homogenized and CFU enumerated in triplicates. The data shown is combined from 2 independent experiments of n = 5 mice per group. The data was analyzed by Mann-Whitney test. Statistical significance was not observed in any of the data.
(TIF)
