# Supplementary figures and images for: Staphylococcal enterotoxin-like X (SElX) is a unique superantigen with functional features of two major families of staphylococcal virulence factors
Source: PLoS Pathog. 2017 Sep 7;13(9):e1006549. doi: 10.1371/journal.ppat.1006549 (PMC5589262; doi:10.1371/journal.ppat.1006549)

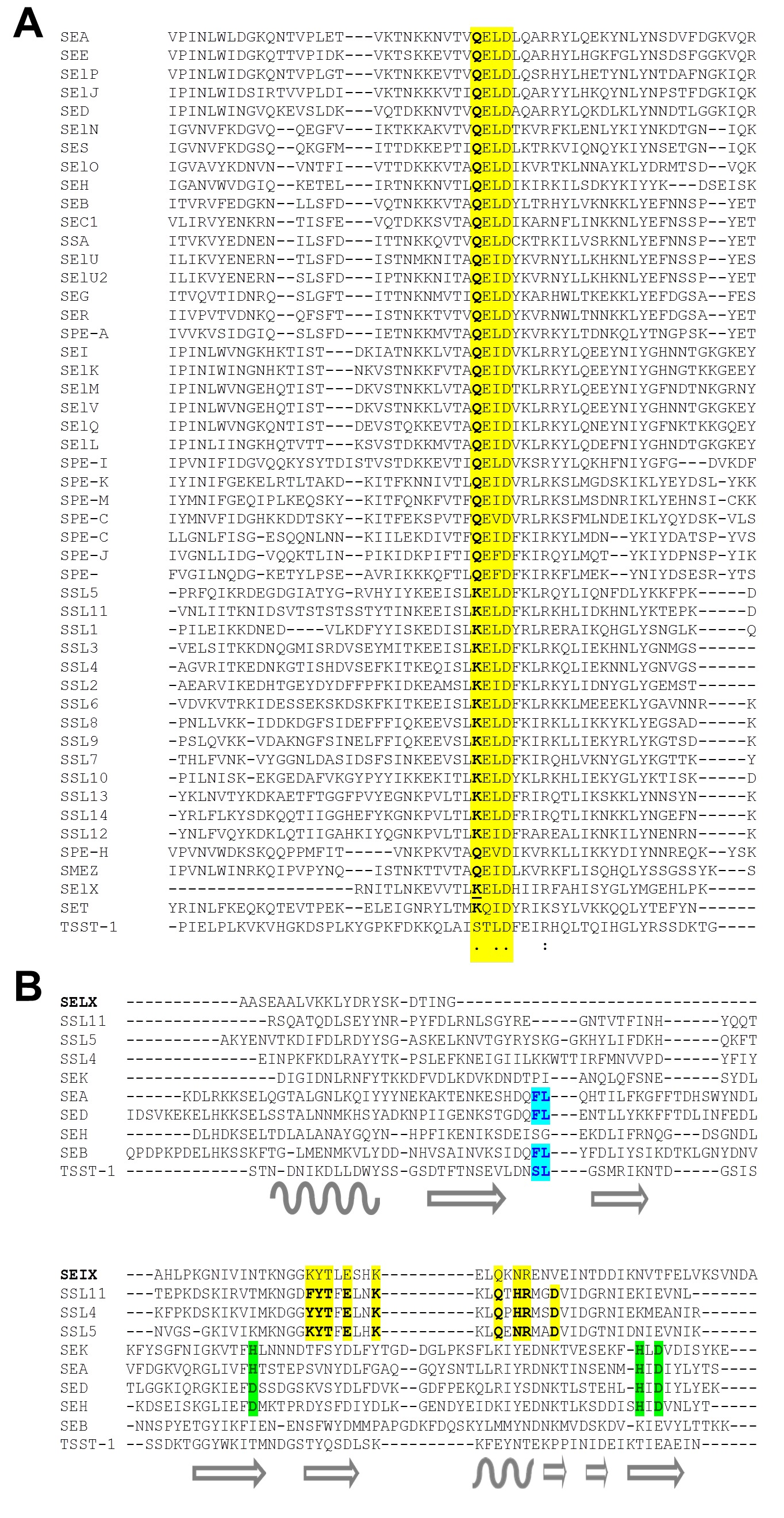

Supplement: S1 Fig — (A) Amino acid alignment of the Group A Streptococcal and Staphylococcal SAgs with the Staphylococcal Superantigen-Like (SSL) proteins in the region of the PROSITE signature sequence PS00278. The consensus sequences of the SSLs [KE(L/I)D] and the SAgs [QE(L/I/V)D] are highlighted in yellow. The Lysine (K) of this motif conserved in the SSLs and the Glutamine (Q) of the SAgs are shown in bold type. (B) Structural alignments of selected S. aureus SAgs and SSLs showing the MHC class II α-chain binding region (upper panel) and MHC class II β-chain binding region (lower panel) generated using PROMALS3D (PROfile Multiple Alignment with predicted Local Structures and 3D constraints) (http://prodata.swmed.edu). Secondary structural elements are shown below the alignments. Amino acids that have been experimentally determined to bind MHC class II are shown highlighted in blue (MHC class II α-chain binding) or green (MHC class II β-chain binding). Amino acids from SSLs that are involved in binding to sialylated glycans are highlighted in yellow. Those that have been determined to bind sLeX by X-ray crystallography are shown in bold type. (TIF) [file ppat.1006549.s002.tif]

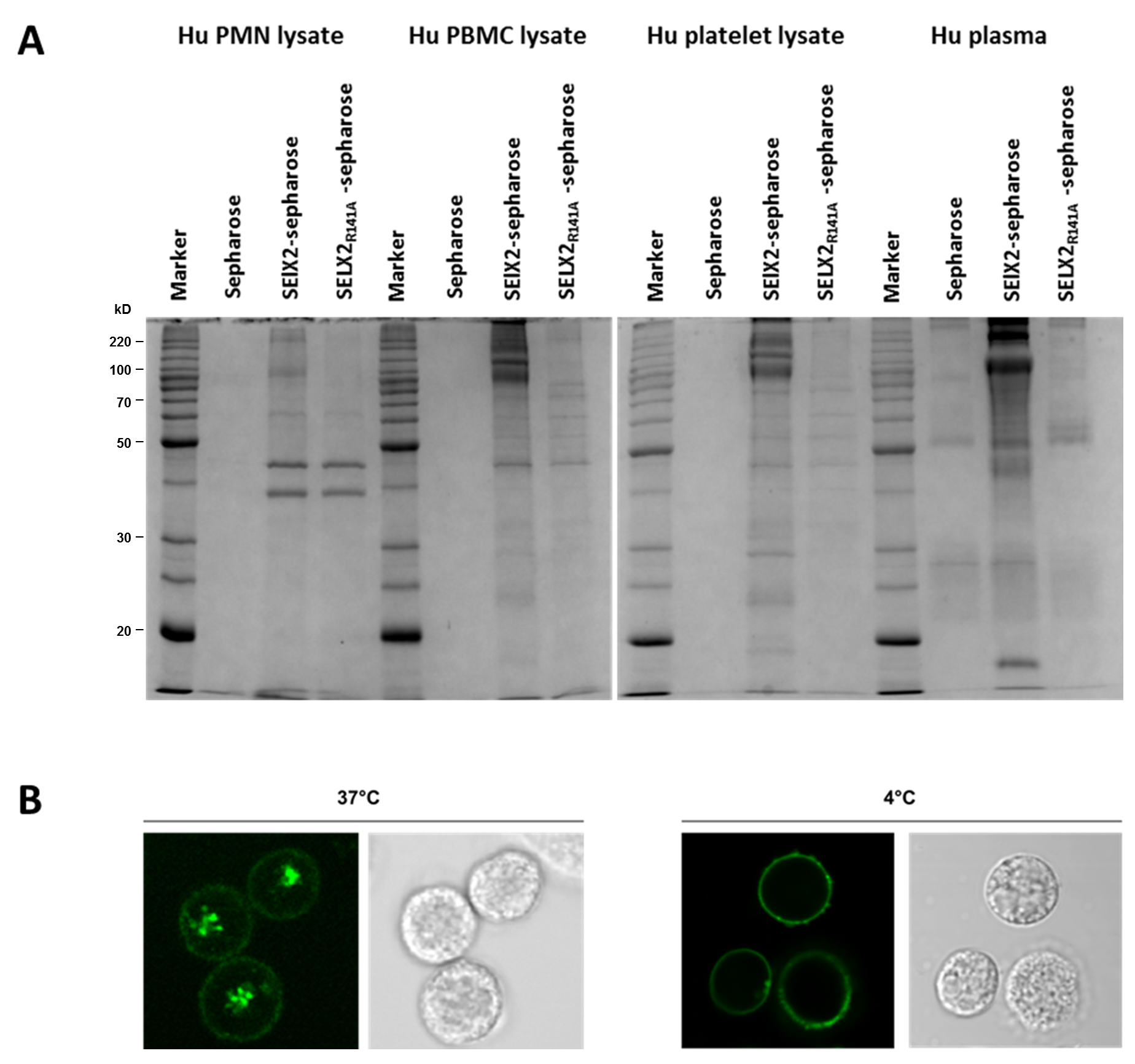

Supplement: S2 Fig — (A) The binding profile comparison of SElX2 and SElX2-R141A. Proteins from cell lysates of human PMN, PBMC, platelets, or from human plasma pulled out of solution by binding to SElX2-sepharose, SElX2-R141A-sepharose, or sepharose alone, and separated by reducing and denaturing SDS-PAGE (12.5%) alongside the Benchmark Protein Marker (Life Technologies). (B) Energy- and sialylated-glycan-dependent binding of SElX to neutrophils. SElX2 conjugated to Alexa Fluor 488 (SElX-488) was incubated with human neutrophils and monitored by live-cell confocal microscopy. After 15 minutes of incubation at 37°C intense and localized intracellular staining of SElX2 similar to that previously described for SSL4 and SSL11 was observed whereas no internalization was seen at 4°C. No cell staining could be seen using fluorescently labelled SElX2-T130A. (TIF) [file ppat.1006549.s003.tif]

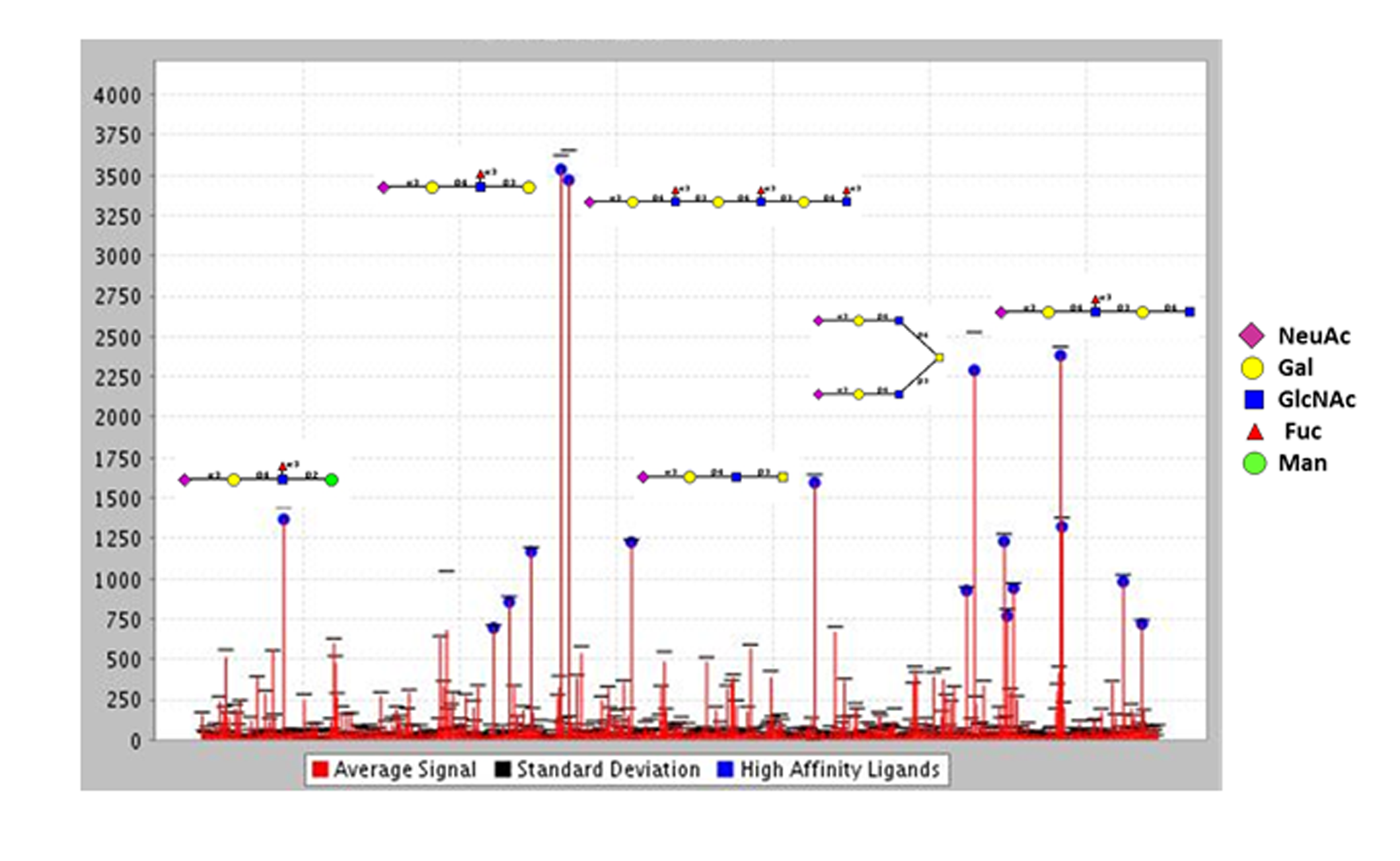

Supplement: S3 Fig — High affinity ligands bound by SElX are labelled with blue circles. The structures of the top binding glycans are shown in cartoon form. These are predominantly structures contain sialyl-lactosamine (sLacNac = Neu5Aca2-3Galb1-4GlcNAc) and sialyl Lewis X (sLeX = Neu5Acα2-3Galβ1-4(Fucα1–3)GlcNAc). N-Acetylneuraminic Acid (NeuAc) purple diamond, galactose (Gal) yellow circle, N-Acetylglucosamine (GlcNAc) blue square, fucose (Fuc) red triangle, and mannose (Man) green circle. (TIF) [file ppat.1006549.s004.tif]

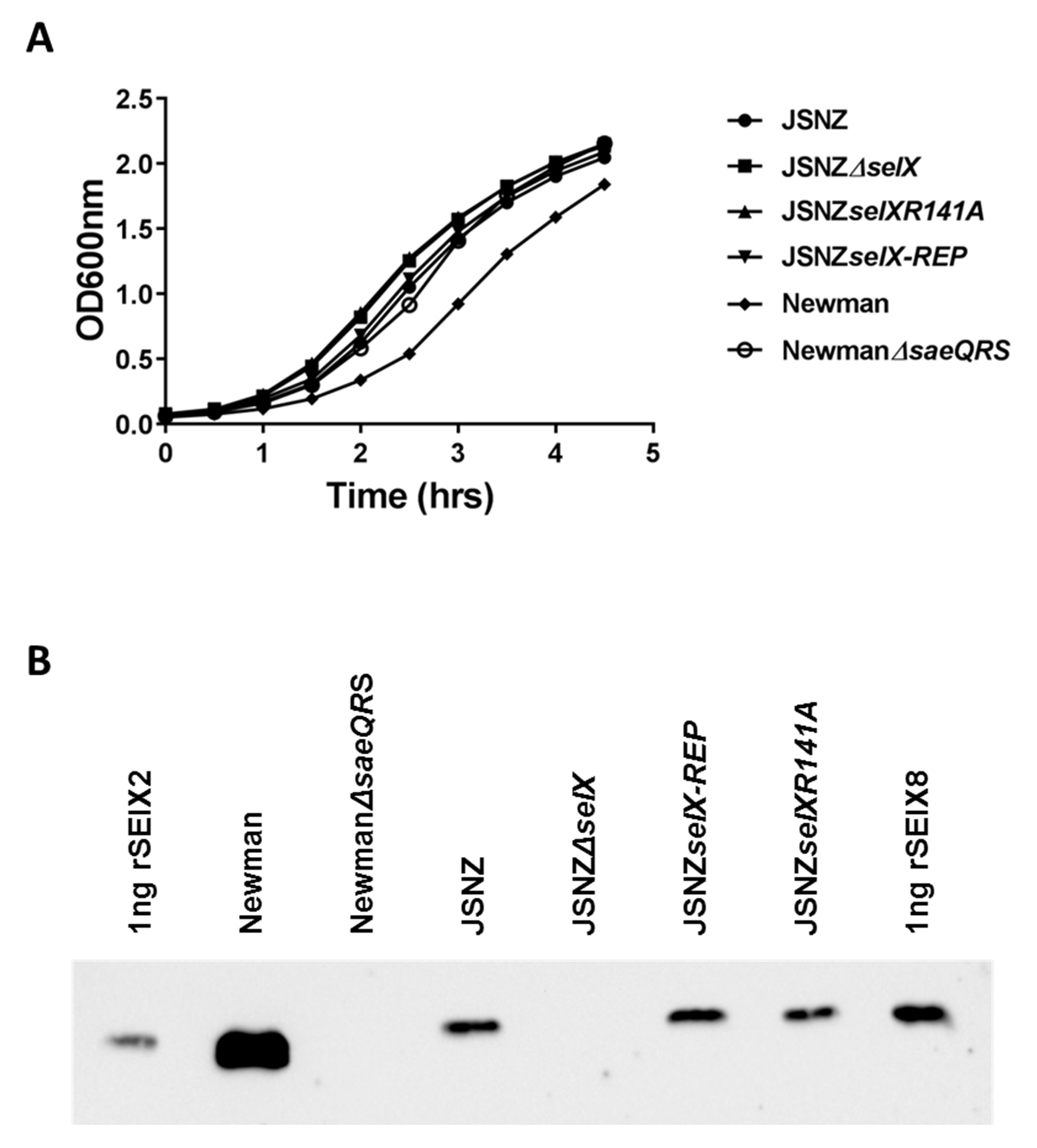

Supplement: S4 Fig — (A) Analysis of the in vitro growth of S. aureus Newman, NewmanΔsaeQRS, JSNZ, JSNZΔselX, JSNZselX-REP, and JSNZselXR141A at 37°C in tryptic soy broth. This data is a representative of two independent experiments and was performed in duplicate. (B) Detection of SElX production by Newman, NewmanΔsaeQRS, JSNZ, JSNZΔselX, JSNZselX-REP, and JSNZselX-R141A. A 5μl sample of culture supernatant from each of the indicated bacteria, growth O/N in RPMI, was, separated by SDS-PAGE (12.5%) alongside 1ng of rSElX2 and rSElX8 included as controls, under reducing and denaturing conditions, and transferred to nitrocellulose. Western analysis was conducted using affinity purified rabbit anti-SElX (made in-house) and the secondary antibody goat anti-rabbit IgG-HRP (AbD serotec). (TIF) [file ppat.1006549.s005.tif]

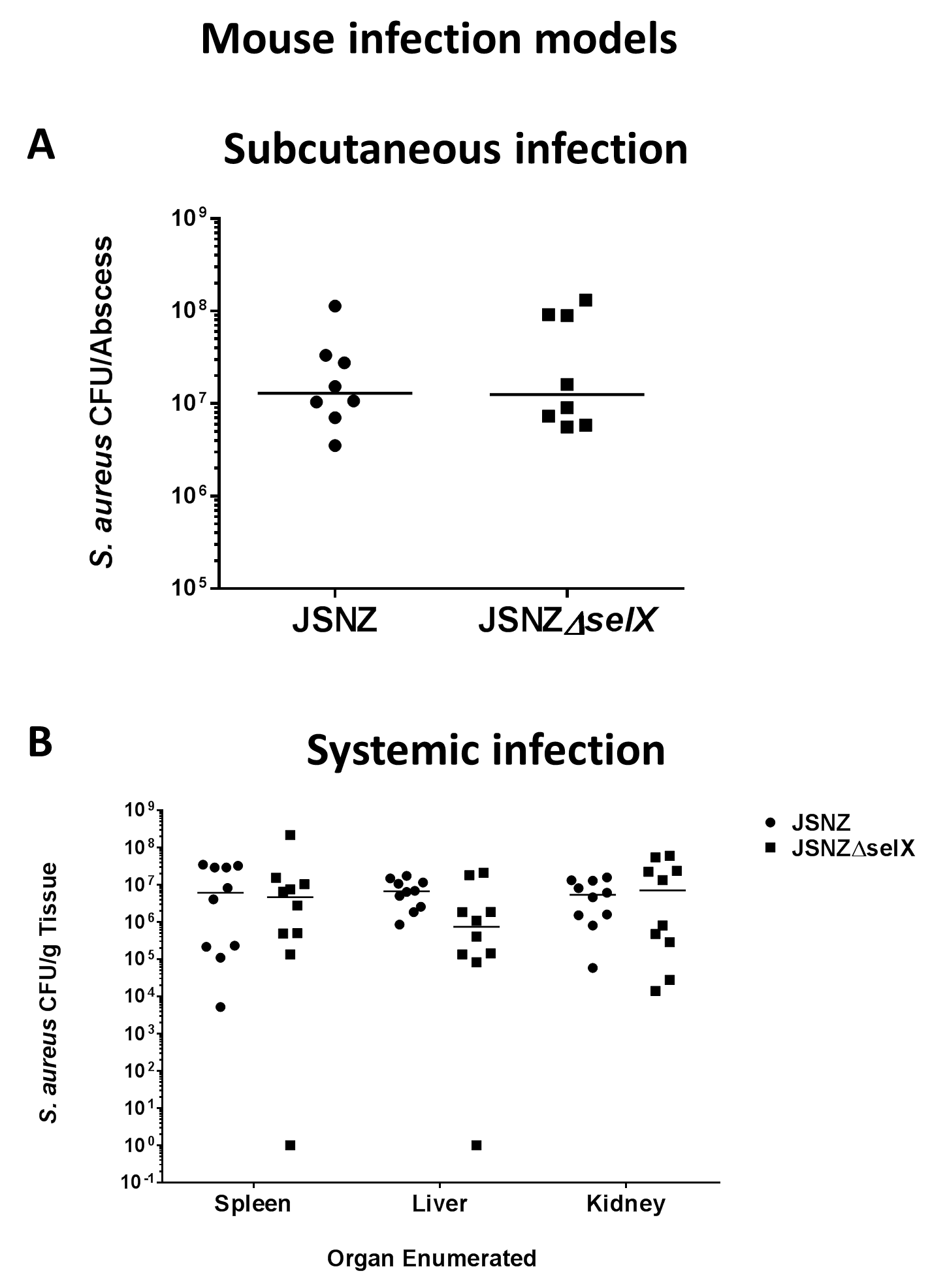

Supplement: S5 Fig — (A) Subcutaneous infection of mice with JSNZ or JSNZΔselX. CD1 mice were injected subcutaneously on the left and right flank with 5 x 10^6^ CFU S. aureus JSNZ or JSNZΔselX. Tissue at the site of infection was removed and homogenised to estimate the CFU/abscess after 96 hours. Each treatment group contained n = 4 mice, each point represents a single abscess and the horizontal bar is the median value. There were no significant differences between the treatment groups (Mann-Whitney). (B) Intrperitineal infection of mice with JSNZ or JSNZΔselX. Mice were intraperitoneally injected with 1 x 10^8^ S. aureus JSNZ or JSNZΔselX. Spleen, Liver and Kidneys from individual mice were removed on day 5 post infection. Samples were homogenized and CFU enumerated in triplicates. The data shown is combined from 2 independent experiments of n = 5 mice per group. The data was analyzed by Mann-Whitney test. Statistical significance was not observed in any of the data. (TIF) [file ppat.1006549.s006.tif]
